# Genome-Wide Methylation Analyses in Glioblastoma Multiforme

**DOI:** 10.1371/journal.pone.0089376

**Published:** 2014-02-21

**Authors:** Rose K. Lai, Yanwen Chen, Xiaowei Guan, Darryl Nousome, Charu Sharma, Peter Canoll, Jeffrey Bruce, Andrew E. Sloan, Etty Cortes, Jean-Paul Vonsattel, Tao Su, Lissette Delgado-Cruzata, Irina Gurvich, Regina M. Santella, Quinn Ostrom, Annette Lee, Peter Gregersen, Jill Barnholtz-Sloan

**Affiliations:** 1 Departments of Neurology, Neurosurgery and Preventive Medicine, University of Southern California, Los Angeles, California, United States of America; 2 Case Comprehensive Cancer Center, Case Western Reserve University, Cleveland, Ohio, United States of America; 3 Department of Radiation Oncology, Columbia University, New York, New York, United States of America; 4 Departments of Pathology, Columbia University, New York, New York, United States of America; 5 Departments of Neurosurgery, Columbia University & Bartoli Brain Tumor Research Laboratory, Columbia University, New York, New York, United States of America; 6 Department of Neurological Surgery, University Hospitals-Case Medical Center, Case Western Reserve University, United States of America; 7 New York Brain Bank, Columbia University, New York, New York, United States of America; 8 Pathology Core, Herbert Irving Cancer Center, Columbia University, New York, New York, United States of America; 9 Department of Environmental Health Sciences, Columbia University & Biomarker Core, Herbert Irving Comprehensive Cancer Center, Columbia University, New York, New York, United States of America; 10 Feinstein Institute of Medical Genetics, North Shore University Hospital, Manhasset, New York, United States of America; CEA - Institut de Genomique, France

## Abstract

Few studies had investigated genome-wide methylation in glioblastoma multiforme (GBM). Our goals were to study differential methylation across the genome in gene promoters using an array-based method, as well as repetitive elements using surrogate global methylation markers. The discovery sample set for this study consisted of 54 GBM from Columbia University and Case Western Reserve University, and 24 brain controls from the New York Brain Bank. We assembled a validation dataset using methylation data of 162 TCGA GBM and 140 brain controls from dbGAP. HumanMethylation27 Analysis Bead-Chips (Illumina) were used to interrogate 26,486 informative CpG sites in both the discovery and validation datasets. Global methylation levels were assessed by analysis of L1 retrotransposon (LINE1), 5 methyl-deoxycytidine (5m-dC) and 5 hydroxylmethyl-deoxycytidine (5hm-dC) in the discovery dataset. We validated a total of 1548 CpG sites (1307 genes) that were differentially methylated in GBM compared to controls. There were more than twice as many hypomethylated genes as hypermethylated ones. Both the discovery and validation datasets found 5 tumor methylation classes. Pathway analyses showed that the top ten pathways in hypomethylated genes were all related to functions of innate and acquired immunities. Among hypermethylated pathways, transcriptional regulatory network in embryonic stem cells was the most significant. In the study of global methylation markers, 5m-dC level was the best discriminant among methylation classes, whereas in survival analyses, high level of LINE1 methylation was an independent, favorable prognostic factor in the discovery dataset. Based on a pathway approach, hypermethylation in genes that control stem cell differentiation were significant, poor prognostic factors of overall survival in both the discovery and validation datasets. Approaches that targeted these methylated genes may be a future therapeutic goal.

## Introduction

Cancers are now recognized as driven as much by epigenetic as well as genetic changes [1]. Among epigenetic alterations that occur during oncogenesis, aberrant gene promoter hypermethylation is the most commonly investigated. However, there have been few studies that evaluated differential promoter methylation across the entire genome in glioblastoma multiforme (GBM), which is the most common type of malignant brain tumors in adults [2]–[5]. The primary goal of some studies, such as the Cancer Genome Altas Project (TCGA), was to characterize methylation patterns in tumors and to correlate with other genomic alterations such as gene mutations, copy number alterations and expression [6]. The investigation of differential methylation poses a challenge, because unlike colon, breast or prostate cancers, it is not possible to obtain matching “normal” tissues during surgery for GBM. The alternative method, which is to procure a substantial number of unrelated normal brain tissues for comparison, is also challenging. Moreover, previous reports on genome-wide methylation in normal brain tissues showed methylation patterns varied between neuro-anatomically distinct regions, and methylation level may change in the brain with increasing age [7]–[9]. Thus, an accurate profile of differential methylation will require appropriate control tissues with age and neuro-anatomical distribution matching those of glioma subjects.

Compared to genome-wide methylation near gene promoters, methylation derangement in the repetitive elements of the GBM genome was even less studied. Repetitive elements may comprise over two-thirds of the human genome, and a high proportion of them are retrotransposons, whose expression is normally suppressed by methylation of cytosine [10]. Retrotransposons become hypomethylated early on in oncogenesis. This can lead to transposable elements insertion, and some of them, such as L1, can express their RNAs, which then promote DNA damage, spreading of methylation to promoters and genomic deletions [11], [12]. Despite their abundance and importance in tumorigenesis, the sequences and maps of repetitive elements in the genome have been difficult to ascertain, because repeats created ambiguities in alignment and in genome assembly [13]. Nevertheless, surrogate markers that estimate global cytosine methylation content, which indirectly reflects methylation levels in repetitive elements due to high CpG contents in those regions (>65% of total genomic CpGs), have been developed and used to study cancer risk, tumor stage, relationship to other molecular phenotypes and prognosis [14]–[18]. One study that measured 5-methyl-cytosine content using a methyl acceptance assay in one epileptic specimen and 10 GBM tissues showed global hypomethylation in tumors [19]. The methylome of other cancers had showed concurrent global hypomethylation and gene promoter hypermethylation [20].

This study had three primary objectives. First, we explored differential methylation of gene promoters/CpG islands across the genome, evaluating more than 14,000 genes at single CpG resolution. To accomplish this goal, we used standard non parametric and biological pathway based analytical approaches to compare primary GBM (de novo) with a substantial number of representative normal brain tissues. Second, we investigated genome-wide methylation level, which included CpG methylation levels in the repetitive elements, as potential diagnostic marker in GBM. We characterized and compared changes in LINE1 (L1 retrotransposon), 5 methyl-deoxycytidine (5m-dC) and 5 hydroxylmethyl-deoxycytidine (5hm-dC). Analysis of LINE1 is widely used as a marker of global cytosine methylation level [10], [18]. Analysis of 5m-dC gives a broader and more accurate measure of global methylation across the genome. 5hm-dC is an oxidized product of 5m-dC generated by the α-ketoglutarate-dependent *TET* dioxygenases [21]. One report showed that 5hm-dC was strongly depleted in glioma and other cancers [21]. Third, we evaluated the prognostic values of methylation pathways and global methylation markers in a multi-variable Cox proportional hazard model, adjusted for *IDH1* mutation, *GCIMP* status, *MGMT* methylation and other clinical factors.

## Materials and Methods

### GBM and Brain Control Tissues in the Discovery Dataset

This study was approved by the institutional review boards (IRBs) of Columbia University (CUMC) and Case Western Reserve University (CWRU). Participants provided written informed consents. Primary GBMs were retrieved from each institution’s biorepositories. All tumor tissues were snap-frozen immediately post resection and were examined neuropathologically. Only tissues with an estimated 80% tumor nuclei and less than 50% necrosis were accepted for DNA extraction and subsequent methylation analyses. Fifty-four (54) de novo GBM (40 from CUMC and 14 from CWRU) passed these criteria and were included in this study.

Control brain tissues were obtained from the New York Brain Bank. We retrieved 24 post-mortem, freshly frozen control tissues from 24 unique individuals. All control tissues had been previously examined by a neuro-pathologist and were verified to be without pathological evidence of other neurological or psychiatric diseases. These 24 brain controls and the aforementioned 54 GBMs comprised the discovery dataset. The data of our discovery dataset was deposited into GEO (accession # 50923).

### GBM and Control Brain Tissues in the Validation Dataset

A validation GBM dataset was retrieved from the publicly available Cancer Genome Atlas Data Portal (TCGA data portal). This dataset comprised of 163 GBM samples submitted at initial diagnoses and were analyzed with the Illumina Methylation27 platform (CWRU cases were excluded). The data were from batches 16, 20, 26, 38 and 62, which were not included in the previous TCGA marker paper on GBM methylation [6]. The four control brain tissues used for that publication were not part of the TCGA dataset and were not available for download in the TCGA Public Portal (personal communication Daniel Weinsberger). Instead, we retrieved 140 publicly available brain tissue controls from GEO accession (# 15745) and dbGAP (phs000249.v1.p1) for comparison with TCGA tumors. This cohort of control brain tissues were obtained from consented subjects, at the time of autopsy, at the University of Maryland, Johns Hopkins University and the National Institute of Aging. They were examined neuropathologically to be without any intra-cranial pathology. The methylation results using Illumina Methylation 27 K platform were published previously [22].

In addition to having a validation dataset, we also validated the most significantly methylated CpG sites via pyrosequencing experiments. We chose those top sites that not only passed our FDR adjusted criteria but also showed at least 4 fold increased or decreased in methylation compared to control tissues. For correlation of validated methylation probes with gene expression, we used the corresponding TCGA gene expression dataset for the same 162 GBM patients (Agilent 244k Custom Gene Expression G4502A-07) to calculate overall Spearman correlation coefficients.

### DNA Methylation and Illumina Infinium Human Methylation 27 K Platform

DNA was extracted by standard proteinase K/RNase treatment and phenol/chloroform extraction. Bisulfite modification of 1 µg of DNA was conducted using an EZ DNA Methylation Kit (Zymo Research, Irvine, CA). The HumanMethylation27 DNA Analysis BeadChips (Illumina) were used to interrogate 27,578 highly informative CpG sites at single nucleotide resolution, covering 14,495 genes. The array hybridization was conducted under a temperature gradient program, and the array was imaged using a BeadArray Reader. Image processing and intensity data extraction was performed as described previously [23].

### Levels of 5m-dC, 5hm-dC and LINE1 Methylation

To determine the overall percentages of 5m-dC and 5hm-dC, we first measured concentrations of dC, 5m-dC and 5hm-dC. We modified a previously published method by adding determination of 5hm-dC levels, using [^15^N_3_]-5m-dC as the internal standard for 5hm-dC and [^15^N_3_]-dC as the internal standard for dC [24]. UPLC/MS/MS positive ionization mode was used to monitor the mass to charge (m/z) transitions of dC: 228.1→112.0; [^15^N_3_]-dC: 231.1→115.0; 5m-dC: 242-1→126.0; [^15^N_3_]-5m-dC: 245-1→129.0 and 5hm-dC: 258.1→142.1. Standard curves were prepared by plotting the analyte/[^15^N_3_]-labeled 5m-dC internal standard ratio (M+0/M+3) against nucleoside concentration, and the concentrations of dC, 5m-dC and 5hm-dC in each sample were calculated. Percentages of 5m-dC and 5hm-dC were obtained by dividing the concentrations of 5m-dC and 5hm-dC by the total concentrations of cytidine nucleosides (dC +5m-dC +5hm-dC).

LINE-1 DNA methylation levels were determined by pyrosequencing as previously described [25]–[27]. Each set of amplifications included bisulfite-converted CpGenome™ (Millipore) universal methylated, unmethylated and non-template controls. Percent methylation within a sample was subsequently determined by averaging across all three interrogated CpG sites. Non-CpG cytosine residues were used as internal controls to verify efficient sodium bisulfite DNA conversion. The inter- and intra-assay coefficients of variation were 1.90 and 1.30%, respectively. All samples were run blinded to tissue status.

### Determination of Glioma CpG Island Methylator Phenotype (*GCIMP*) Status

In both the discovery and validation datasets, we found those probes in the Illumina 27 K array that corresponded to the validated GCIMP markers as documented by Noushmehr et al [6]. They were ANKRD43, HFE, MAL, LGALS3, FAS, RHO-F and DOCK5. Although the paper documented 2 FAS markers: FAS-1 and FAS-2, they represented different regions of the promoter of FAS gene in the MethylLight assay only (personal communication, Daniel Weisenberger). The 27 K array contained only one probe for FAS. Thus our clustering analyses used 7 instead of 8 markers to identify those GCIMP+tumors. In the TCGA validation dataset, we also verified our list of GCIMP+tumors with those reported in the NCI TCGA Wiki, which maintained records of genomic analyses of GBM.

### *IDH1* Mutation

For GBM samples in the discovery dataset, IDH1 mutation status was determined via pyrosequencing. The portion of IDH1 spanning codon 132 (75 bp amplicon) was amplified. Forward primer was 5′-GCTTGTGAGTGGATGGGTAAA-3′ and biotinylated reverse primers was 5′-TTGCCAACATGACTTACTTGATC-3′. Polymerase chain reaction (PCR) and pyrosequencing assays were performed as previously described [28]. Pyrosequencing primer provided sequence data that included codon 131 and the first nucleotide of codon 132 (5′-GGGTAAAACCTATCATCATA-3′). Negative controls were run with all subjects’ samples. Sequence data were analyzed using PyroMark Q24 software.

For GBM samples in the TCGA validation dataset, we determined their *IDH1* mutation status by examining their level 2 DNA sequencing data, which was generated using Illumina’s Genome Analyzer (GA).

### *MGMT* Methylation

For *MGMT* methylation status in the discovery dataset, we chose pyrosequencing as the analytical method, because previous studies showed that it provided the best prognostic value, cost effectiveness and ease of use [29]. Seven CpG sites in the promoter region of *MGMT* were selected based on previous validations, with a Qiagen kit (PM00149702) [30]. Polymerase chain reaction (PCR) was performed in a 25-ul reaction mix containing 50 ng of bisulfite-converted DNA, 1x Pyromark PCR Master Mix (Qiagen), 1x Coral Load Concentrate (Qiagen), and 0.3-uM forward and 5′ biotinylated reverse primers, using the cycling conditions and amplifications as outlined previously [31]. Each set of amplifications included bisulfite-converted CpGenome™ universal methylated (Millipore, Billerica, MA), unmethylated (whole genome amplified DNA), and non-template controls. The sequencing reaction and quantitation of methylation was conducted using a PyroMark Q24 instrument and software (Qiagen). Percentage methylation was calculated by averaging across all CpG sites interrogated. As percentage methylation is a continuous variable, we converted it to a binary variable using a “cutoff” to facilitate clinical interpretation. There has been no established consensus cut-off for pyrosequencing percentage values, but in normal brain tissues, average MGMT promoter methylation ranges between 0% and 10% [29]. Thus, as reported previously, we used 14% as the threshold to distinguish unmethylated from methylated *MGMT* promoter in a given tumor [15].

Since TCGA did not separately provide *MGMT* methylation level of their GBMs, we used the CpG probes on the Illumina 27 K array to determine these tumors’ *MGMT* methylation status. Two validated *MGMT* probes, cg12434587 and cg12981137, were used in prognostic models as a continuous variable, because a previous study confirmed their prognostic and classification properties [32].

### Pyrosequencing Validation of Differentially Methylated Genes

For other significant CpG sites that were differentially methylated, the regions selected for interrogation covered the particular CpG sites on the Illumina arrays as well as surrounding sites. PCR and pyrosequencing assays were as described above using Qiagen kits. Primers were included in Table S1. Percent methylation of each gene was calculated by averaging across all CpG sites interrogated.

### Statistical Methods

#### Data assembly

Each methylation data point represents the fluorescent signals from the M (methylated) and U (unmethylated) alleles. Background intensity was computed from a set of negative controls and was subtracted from each analytical data point. The ratio of fluorescent signals was then computed from the 2 alleles to reflect the fractional methylation level at each CpG site (β-value), which is between 0 and 1 as the proportion of methylation for a given CpG site. Beta values were generated using Illumina BeadStudio software. For quality control, methylation measures with a detection P value >0.05 and samples with CpG coverage <75% were removed (for 7 probes total). All X and Y chromosome probes (including 1,085 in X and 7 in Y) were dropped, leaving 26,486 probes for all further analyses. We performed two major types of analyses: 1. Locus by locus comparison between GBM and control brain tissues; 2. Unsupervised hierarchical clustering of tumors and control tissues.

#### Locus-by-locus analyses

For both the discovery and validation datasets, we first filtered out those CpG sites with median |Δβ| <0.2, as studies in the past had shown that this methylation array cannot accurately detect β difference at or below 0.17 [23]. Then we used the non-parametric Wilcoxon Rank Sum test to compare each CpG site’s methylation levels between normal brain tissues and controls; Benjamin-Hochberg false discovery rate (FDR) was used to adjust for multiple comparisons. Significance level was set at FDR ≤0.05. Due to influence on differential methylation by neuroanatomical region and age, we also performed an adjusted analysis using a published method based on logistic regression: logit (P) = µ_ij_+A*β_ij_+B*age_j_+C*location_j_+ε_ij_, where P is the probability to be a tumor; β = beta for the CpG probe i of sample j; μ = intercept for the CpG i of sample j; age = age of the patient from sample j; location = brain location of sample j; ε = error term of the CpG i of sample j [33]. Histograms were generated to show median |Δβ| distributions of hyper- and hypomethylated CpG sites.

#### Unsupervised hierarchical clustering

To explore data patterns, we performed unsupervised hierarchical clustering on those differentially methylated CpG sites (FDR ≤0.05) using Euclidean distance metric and Ward linkage. The same clustering algorithm was applied to the discovery and validation datasets. To further reduce the dimensionality of our datasets, we also used principal component analyses (PCA) with correlation matrix for data reduction.

#### Biological pathways involved in differential methylation

Ingenuity Pathway Analysis (IPA, Ingenuity Systems, Redwood City, CA) was used for canonical pathway analyses of those validated, differential genes. This bioinformatics tool was used to provide insights into the most involved biological pathways in tumorigenesis based on DNA methylation alterations.

#### Correlation of global methylation markers with methylation classes

We compared LINE1, 5m-dC and 5hm-dC levels among methylation classes using the non-parametric Kruskal-Wallis Test, as prior analyses had shown that these markers were not normally distributed [26]. Post-hoc pairwise comparisons after significant Kruskal-Wallis Tests were conducted using the Tukey HSD test.

#### Survival analyses

Prognostic assessments were performed separately for the discovery tumor dataset and the TCGA validation dataset, using Cox proportional hazard regression models. We investigated the value of methylation biomarkers other than *MGMT* or *GCIMP*, such as LINE1, 5m-dC and 5hm-dC, as potential independent prognostic factors. Moreover, biological processes do not act through the effect of a single gene but are the results of combined influences of many genes in a relevant pathway. Thus we also explored the net effect of the most important methylated pathways, such as those discovered by IPA, as prognostic factors. To achieve this goal, we calculated *an index* for a top pathway, which is a combination of the cross product of beta values and univariable Cox regression coefficient of each involved genes in that pathway. The index for the pathway was then evaluated in regression models. This method was previously published in other pathway-based survival studies using genome-wide microarrays [34]. Standard clinical and molecular pathology information included *MGMT* methylation, *GCIMP* status, *IDH1* mutation, age at diagnoses, Karnofsky performance score (KPS) at the time of diagnoses, extent of surgical resection, bevacizumab use at recurrence or tumor progression and the center which provided the specimens. In the discovery dataset, concomitant chemo-radiation was not included in survival model, as all patients received combined treatment. In the TCGA dataset, there was no information on extent of surgery and no global methylation biomarkers.

Each prognostic factor was first evaluated in a univariable Cox proportional hazard model analyses. Those factors that reached significance levels of p≤0.1 were entered into the multivariable Cox model. All prognostic factors in the multivariable model were then removed one by one via backward elimination if the covariate p value is >0.05. This process continued until covariates kept in the model were all significant. Then the eliminated factors were added one-by-one back into the model to ensure that they were not significant in the multivariable model. The final model consisted of all significant factors (p≤0.05) in the presence of each other. For each covariate, proportional hazard assumption was tested by plotting scaled Schoenfeld residuals against the natural log of time.

## Results

### Demographics of GBM Cases and Normal Brain Controls

The clinical, demographic and pathological characteristics of GBM cases and brain controls are detailed in Table 1. Most GBM and control tissues came from subjects over age 50. Controls from GEO/dbGAP had median age younger than other groups. More men than women were represented in both tumors and controls. Frontal, parietal and temporal lobes represented the most common anatomical locations of both tumors and control tissues. For the group of brain controls retrieved from GEO/dbGAP, methylation data from four brain locations: frontal, temporal, pons and cerebellum were available. We only used frontal and temporal brain controls for comparison with TCGA glioblastoma, because these tumor tissues were mostly from frontal and temporal lobes. For NYBB brain controls, the frozen postmortem interval (PMI), which was calculated from the subject’s reported time of death to the time the brain was processed, was a median of 5 hours; this time interval was shorter than that of 14.5 hours of dbGAP brain controls or other post-mortem brain tissues [8], [9]. Causes of death for NYBB controls were cardiac (n = 14), pulmonary (n = 4), renal (n = 2), trauma (n = 2), cholangiocarcinoma (n = 1; no brain metastases) and unknown (n = 1). The causes of death for GEO/dbGAP controls were unknown.

**Table 1:** Demographic, clinical and pathological characteristics of subjects in this study.

Demographic/pathological & clinical features	Discovery GBM (n = 54)	TCGA∧ GBM (n = 162)	NYBB* controls(n = 24)	Publicly available controls (n = 140)#
**Age** (median, IQR*)	57 (52–66)	59 (50–67)	67 (57–80)	43 (27–59)
**Women** (%)	22 (40.74)	64(39.51)	11(45.83)	44 (31.43)
**Ethnicity** (%)				
Caucasian	48 (88.89)	145 (89.51)	22 (91.67)	140 (100)
African American	2 (3.70)	9 (5.56)	2 (8.33)	0 (0.00)
Hispanic	2 (3.70)	0 (0.00)	1 (4.17)	0 (0.00)
Oriental	2 (3.70)	3 (1.85)	0 (0.00)	0 (0.00)
Unknown	0 (0.00)	5 (3.09)	0 (0.00)	0 (0.00)
**Anatomical location** (%)				
Frontal	23 (42.59)	51 (31.48)	8 (33.33)	70 (50.00)
Parietal	10 (18.52)	27 (16.67)	7 (29.17)	0 (0.00)
Temporal	11 (20.37)	4 (26.54)	4 (16.67)	70 (50.00)
Occipital	6 (11.11)	10 (6.17)	2 (8.33)	0 (0.00)
Insula	1 (1.85)	0 (0.00)	2 (8.33)	0 (0.00)
Cerebellum	1 (1.85)	0 (0.00)	1 (4.17)	0 (0.00)
Midbrain/Pons/Medulla	1 (1.85)	0 (0.00)	0 (0.00)	0 (0.00)
Basal Ganglia	1 (1.85)	1 (0.62)	0 (0.00)	0 (0.00)
Missing information	0 (0.00)	30 (18.52)	0 (0.00)	0 (0.00)
**Postmortem interval** (hours) (median, IQR*)	NA	NA	5 (4.00–8.50)	14.5(10.00–18.00)
**Clinical information**				
**Surgery (%)**		NA	NA	NA
Biopsy	2 (3.70)			
Subtotal resection	20 (37.04)			
Gross total resection	32 (59.26)			
KPS score (median, IQR*)	80 (70–90)	80 (60–80)		
**Concomitant radiation with** **Temozolomide (%)**			NA	NA
None	0 (0.00)	46 (28.40)		
Received combined therapy	54 (100.00)	107 (66.05)		
Information missing	0 (0.00)	9 (5.56)		
**Treatment with Bevacizumab at disease progression/recurrence (%)**			NA	NA
No	25 (46.30)	114 (70.37)		
Yes	29 (53.70)	38 (23.46)		
Missing	0 (0.00)	10 (6.17)		

*IQR = interquartile range; NYBB = New York Brain Bank.

∧TCGA GBM cases did not include CWRU TCGA GBM cases.

# Publicly available brain tissue controls were from Brain Banks at the University of Maryland, Johns Hopkins University and National Institute of Aging.

### Differential Methylation between GBM and Control Brain Tissues in the Discovery Dataset

**Figure 1:**
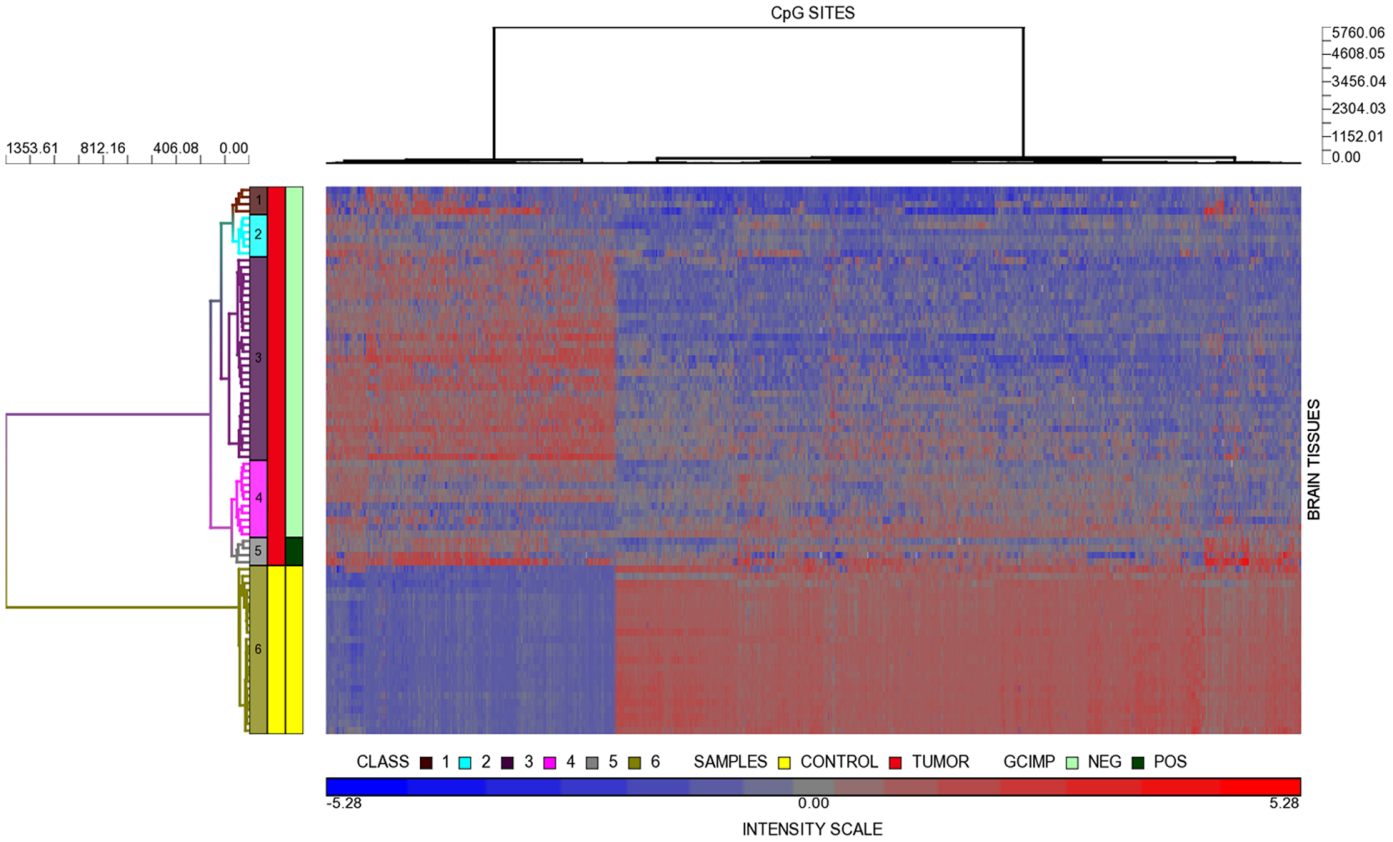
Heat map of differential methylation in the discovery dataset. Heat map based on a set of 1864 CpG sites that significantly segregated GBM and control brain tissues into six methylation classes in the discovery dataset. Methylation class numbers are marked inside the annotation bar. The heat map columns represented CpG probes, and the rows are tumor and control brain samples. In the color scale, relative hypermethlyation is denoted by a shift towards the red color, and relative hypomethylation towards blue. Neutral methylation is gray.

Methylation in 1864 CpG sites, corresponding to 1639 genes, differed significantly between GBM and normal brain tissues in the discovery dataset. Unadjusted and adjusted analyses essentially gave the same CpG list. Table S2 shows the complete list of unadjusted, differentially methylated CpG sites in the discovery dataset. Overall 1389 CpG sites (1175 genes) were hypomethylated in the tumors relative to controls, and 475 CpG sites (464 genes) were hypermethylated. The top 10 most differentially hypomethylated and hypermethylated CpGs are presented in Table 2 and 3, respectively. Figure 1 shows two key features of the unsupervised hierarchical clustering analysis. First, tumors and controls segregated into six classes, with five classes of tumors and one of control. Control brains did not form subgroups based on age or tissue of origin. Class 3 is the dominant tumor class with 29 subjects. Class 5 contained four tumors that were positive for the Glioma CpG Island Methylator Phenotype (*GCIMP+*). *GCIMP* status was verified using the markers described by Noushemir et al (Figure S1a). As previously reported, subjects with *GCIMP+* tumors were significantly younger than *GCIMP–* subjects (p = 0.007). Pyrosequencing analyses showed that five tumors harbored mutations in *IDH1*: four R132H and one R132L mutations. Four of these five *IDH1* mutated tumors corresponded to the four *GCIMP+*GBMs but one was a *GCIMP* negative tumor.

**Table 2:** The top 10 most differentially hypomethylated genes in the discovery dataset.

Genesymbol*	GenomicLocation	Biological Function*	Median ofnormalmethylationlevels (β)	Median oftumormethylationlevels (β)	Median βdifference	P-value	FDRadjustedP value
*LTC4S*	5q35.3	Production of leukotrienesfrom arachidonic acid	0.40	0.16	−0.24	8.20E-07	0.001
*TNFRSF1A*	12p13.31	Receptor for TNF; activateNF-kappaβ	0.32	0.06	−0.26	1.00E-06	0.001
*TMEM71*	8q24.22	Transmembrane protein localizedto the ER with unknown function	0.31	0.09	−0.23	2.20E-06	0.001
*CCL8*	17q12	Mediates chemotactic activityfor leukocytes	0.33	0.12	−0.21	2.30E-06	0.001
*PYGM*	11q13.1	Enzyme involved inglycogenolysis	0.40	0.11	−0.30	2.40E-06	0.001
*PDCD1LG2*	9p24.1	Regulates activated T cellfunctions	0.48	0.17	−0.31	2.50E-06	0.001
*PPP1R3B*	8p23.1	Regulates glycogen synthesis	0.38	0.13	−0.24	2.60E-06	0.001
*GUCY2D*	17p13.1	Membrane guanylyl cyclases	0.40	0.09	−0.31	2.70E-06	0.001
*MMP14*	14q11.2	Activates MMP2 and mediatesoncogenesis	0.39	0.09	−0.30	2.80E-06	0.001
*WNT4*	1p36.12	Involves in inflammation,development and oncogenesis	0.44	0.21	−0.23	2.90E-06	0.001

*LTC4S: leukotriene C4 synthase; TNFRSF1A: tumor necrosis factor receptor superfamily, member 1A; TNF: tumor necrosis factor; TMEM71: encoding transmembrane protein 71; ER: endoplasmic reticulum; CCL8: chemokine ligand 8; PYGM: phosphorylase, glycogen, muscle; PPP1R3B: protein phosphatase 1, regulatory subunit 3B; GUCY2D: guanylate cyclase 2D; MMP14: matrix metallopeptidase 14; WNT4: wingless-type MMTV integration site family, membrane 4.

**Table 3:** The top 10 most differentially hypermethylated genes in the discovery dataset.

Genesymbol*	GenomicLocation	Biological Function	Median ofnormalmethylationlevels (β)	Median oftumormethylationlevels (β)	Median βdifference	P-value	FDRadjustedP value
*MTSS1*	8q24.13	A putative tumor suppressorgene in cancers	0.49	0.90	0.41	1.10E-06	0.001
*LDB3*	10q23.2	A PDZ domain containingprotein that regulatesion channels	0.55	0.87	0.32	1.50E-06	0.001
*HIPK2*	7q34	Interacts with multipletranscription factors	0.39	0.69	0.30	1.60E-06	0.001
*PKD2*	4q22.1	Involves in calciumtransport and signaling	0.55	0.80	0.26	2.30E-06	0.001
*C11orf39*	11q25	Function unknown	0.55	0.84	0.29	4.60E-06	0.001
*Ells1*	7p14.3	Involves in lysineubiquitylation andproteasomal degradation	0.27	0.67	0.40	4.90E-06	0.001
*C11orf2*	11q13	Involves in steroidmetabolism	0.68	0.90	0.22	6.10E-06	0.001
*FLJ36268*	9p22.2	Located in a common fragilesite; over-expressionmay lead to genomicinstability	0.51	0.79	0.28	6.40E-06	0.001
*ZNF146*	19q13.1	A Kruppel protein thatregulates telomere	0.57	0.79	0.22	7.50E-06	0.001
*GUP1*	3p22.1	Negatively regulatesN terminal proteinpalmitoylation	0.43	0.75	0.32	8.20E-06	0.001

*MTSS1: metastasis suppressor 1; LDB3: LIM domain binding 3; HIPK2: homeodomain interacting protein kinase 2; PKD2: polycystic kidney disease 2; C11orf39: chromosome 11 open reading frame 39; Ells1: chromosome 7 open reading frame 41; C11orf2: chromosome 11 opening reading frame 2; FLJ36268: chromosome 9 open reading frame 139; ZNF146: zinc finger protein 146; GUP1: hedgehog acyltransferase-like.

**Figure 2:**
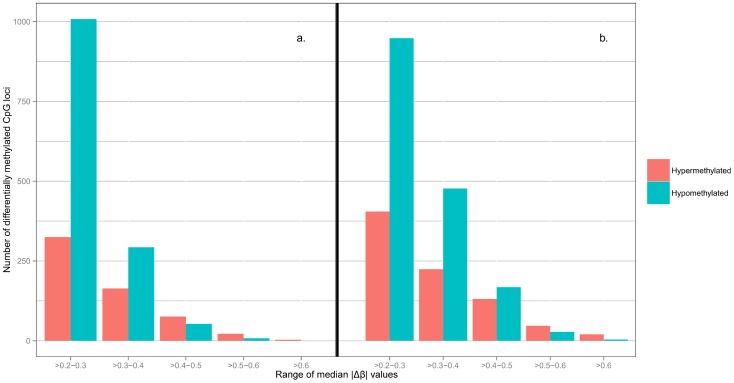
Histograms of median |Δβ| distributions. a. The histogram showing a range of median |Δβ| of significant CpG sites from the discovery dataset; b. the histogram of a range of median |Δβ| of significant CpG sites from the validation dataset. Red bars denote the number of hypermethylated CpGs, and blue bar represented hypomethylated CpGs.

The second feature of the heatmap showed that hyper-and hypomethylated CpG sites separated well into two respective blocks (see columns in Figure 1). Overall, nearly 70% differentially methylated probes were relatively hypomethylated in the tumors. Among tumor classes, each class showed variations in the pattern or degree of hypo and hyper methylation. Class 1 tumors appeared to have higher degree of hypomethylation than other tumor classes. Class 3 tumors showed the clearest transition from hypermethylated to hypomethylated CpG blocks. Compared to brain controls, Class 5 (*GCIMP+*) is only hypermethylated at discrete loci. Figure 2a illustrates the range of values of median |Δβ| in the discovery dataset. There were more hypomethylated than hypermethylated CpGs at moderate |Δβ| between 0.2 and 0.49; however, hypermethylated CpGs predominated when |Δβ| >0.5.

**Figure 3:**
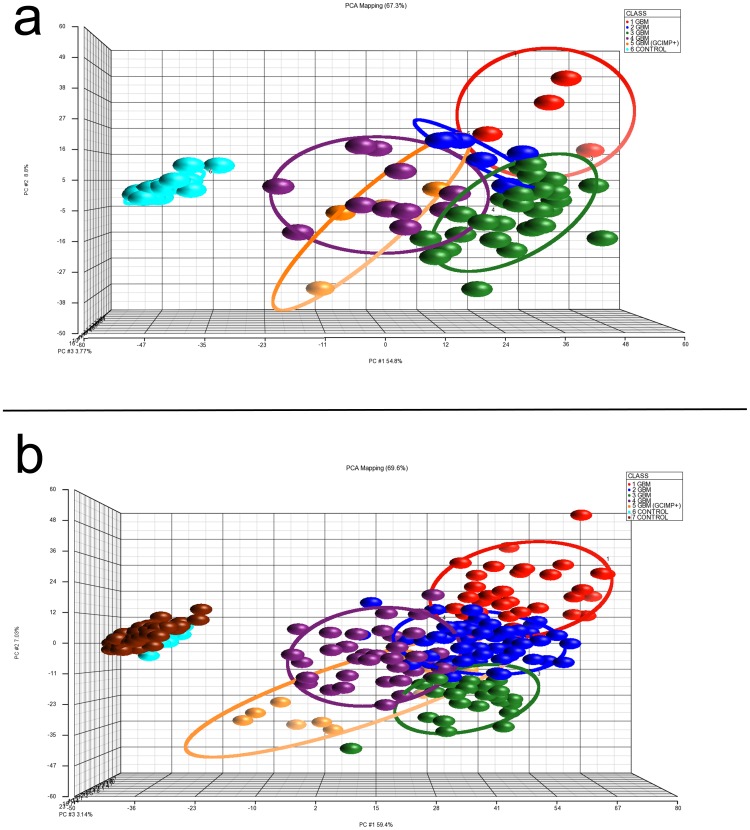
Principal component analyses (PCA) of the discovery and validation datasets. a. In the discovery dataset, the 5 tumor and 1 control methylation classes were represented by the first 3 principal components (PCs) in x, y and z axes in 3 dimensional space. b. In the validation dataset, 5 tumors and 2 control classes were represented by the first 3 PCs in 3 dimensional space.

Figure 3a illustrates principal component analyses (PCA) of the discovery dataset. The 1864 significant CpGs can be reduced to 40 orthogonal principal components (PC) that explained 95% of the variance of the dataset, with the first three PC explained 67% of the variance. Overall, controls clustered tightly together, whereas GBM showed wide dispersion in space due to increase in tumor variance. Each of the 5 tumor classes had its own elliptical plane that is orthogonal to each other, though some members of the classes overlapped each other at the periphery. Figures S2a shows the top down view of PCA analyses. It illustrates the posterior position of the GCIMP+ group, which was apart from other methylation classes but was difficult to fully appreciate from the frontal view.

Table 4 shows the correlations in methylation level between *MGMT* and 5 differentially methylated CpG sites from the discovery dataset using Illumina’s BeadChip and pyrosequencing assays. Correlations overall using Spearman’s *rho* statistics was >0.8. Thus our results supported previous reports of excellent validations of this methylation array technology using pyrosequencing [3], [31].

**Table 4:** The correlations between β values of CpG sites and corresponding mean percentage methylation levels using pyrosequencing from our discovery dataset.

Gene Symbol	Mean pyrosequencinglevel (%)	Mean methylation level (β)	Spearman’s Correlation (ρ)	P-value
*MGMT*	31.98	0.64	0.51	<0.00001
*BHMT*	55.51	0.63	0.93	<0.0001
*BST2*	33.45	0.26	0.94	<0.0001
*DAB2IP*	5.90	0.27	0.80	<0.0001
*DGKE*	42.84	0.49	0.97	<0.0001
*PCDHGB4*	35.38	0.41	0.88	<0.0001

### Results of Validation Dataset and Pyrosequencing Validation

**Figure 4:**
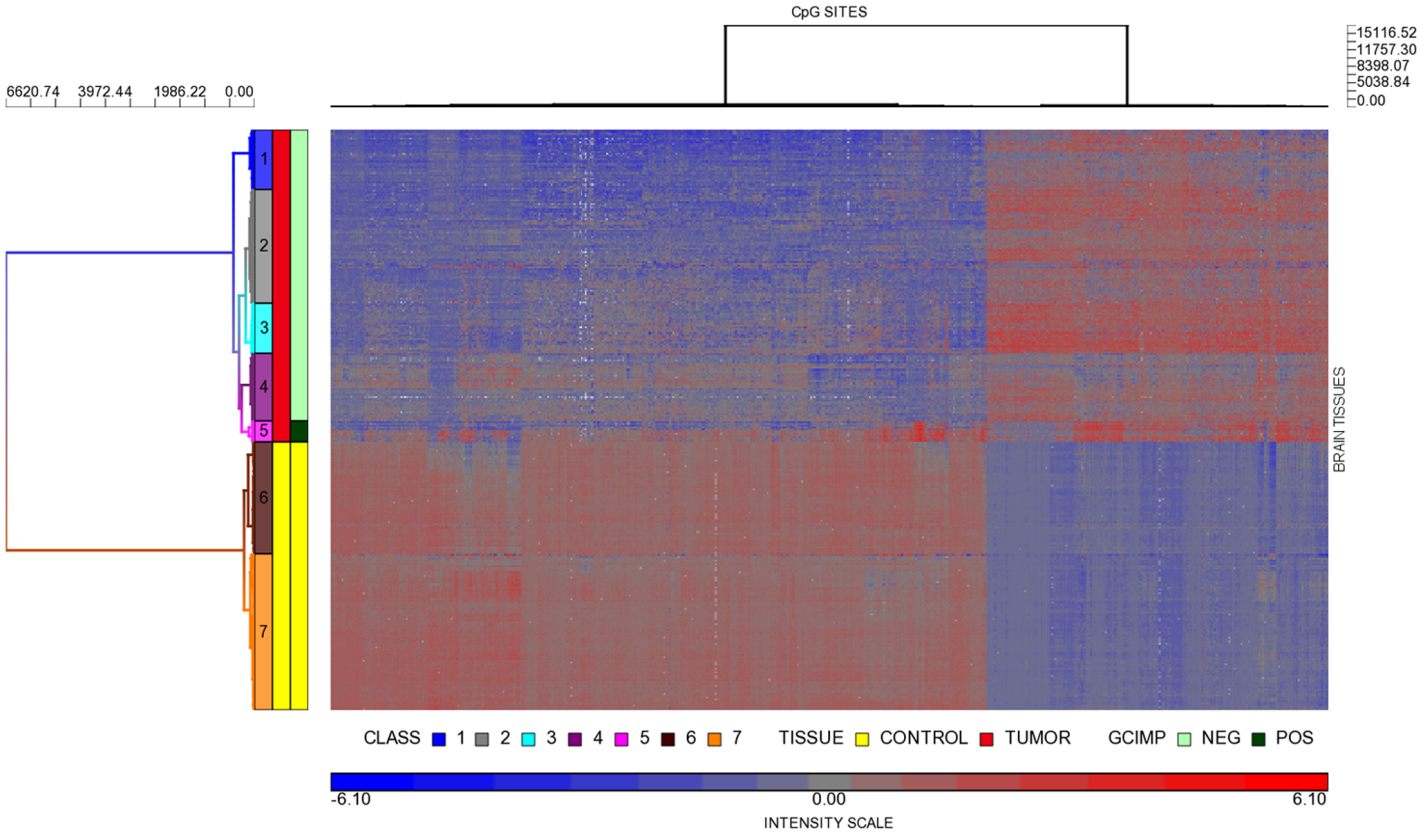
Heat map of differential methylation in the validation dataset. Heat map based on a set of 2445 CpG sites that significantly segregated GBM and control brain tissues into seven methylation classes in the validation dataset. Annotations were the same as Figure 1.

Comparison of 163 TCGA GBMs with 140 publicly available controls showed 2445 CpG sites (2018 genes) were differentially methylated between GBM and normal. Table S3 shows the list of differentially methylated CpGs. There were 1625 hypomethylated CpGs (1368 genes) and 820 hypermethylated CpG sites (650 genes). Figure 4 shows the heatmap of the validation dataset, which also demonstrates hyper and hypomethylated probes formed two separate blocks. Tumors and controls were clustered into 7 *classes*. Similar to the discovery dataset, there were 5 classes of tumors but 2 of controls. Control subjects in Class 6 were significantly older than those in Class 7 (p<0.03). Class 1 tumors contained CpG sites that acquired a higher degree of hypomethylation. Class 5 had 13 GBMs that were *GCIMP +*. Again, relative to controls, hypermethylated CpGs were located in discrete loci. Figure S1b showed unsupervised hierarchical clustering that identified these 13 *GCIMP+*tumors, using markers as described by Noushemir et al. Our results were also confirmed by those reported in the TCGA Wiki. Out of these 13 *GCIMP+* tumors, 6 were *IDH1* mutated. Similar to the GBMs in our discovery dataset, the distribution of median |Δβ| ranges showed hypomethylated CpGs were more prevalent in the moderate |Δβ| range. But at |Δβ| >0.5, there were more hypermethylated probes (Figure 2b).

Figure 3b shows the PCA analysis of the validation dataset, which reduced 2445 significant CpGs to 112 orthogonal PC that explained 95% variance of the dataset, and the top 3 PC accounted for 69% of the total variance. Similar to our discovery dataset, controls clustered tightly together, whereas GBM showed a wide range of methylation variability. Supporting Figure S2b shows the top down view of the PCA analyses, which also illustrates the posterior and separate position of the GCIMP+ group. The physical relationship between the two control groups is better visualized in this view as well.

Overall, 1548 CpG sites (1307 genes) in the validation dataset overlapped with the 1864 CpG sites (1639 genes) from the discovery dataset (83%). Of the 1307 validated genes, 905 were hypermethylated and 402 were hypomethylated. These differentially methylated CpGs and corresponding genes are included in Table S4.

Out of 1307 validated, differentially methylated genes, 1130 had available matched mRNA expression in the TCGA GBM data files (Agilent 244k Custom Gene Expression G4502A). All 163 TCGA GBM cases had corresponding methylation and mRNA expression data. The overall Spearman’s rho was −0.42 (95 CI −0.54, −0.29, p-values = 0.018) for hypermethylated genes, and −0.28 (95% CI −0.42, −0.13, p value = 0.043). In the set of hypermethylated genes, 71. 89% of methylation-expression pairs showed significant inverse correlations (p≤0.05), whereas 55.36% of hypomethylated genes-expression pairs were inversely related. Hence, gene expression and methylation intensity were negatively correlated for these significant genes, but correlation appeared to be stronger for hypermethylated genes.

### Biological Characteristics of Validated Genes and Involved Pathways

**Figure 5:**
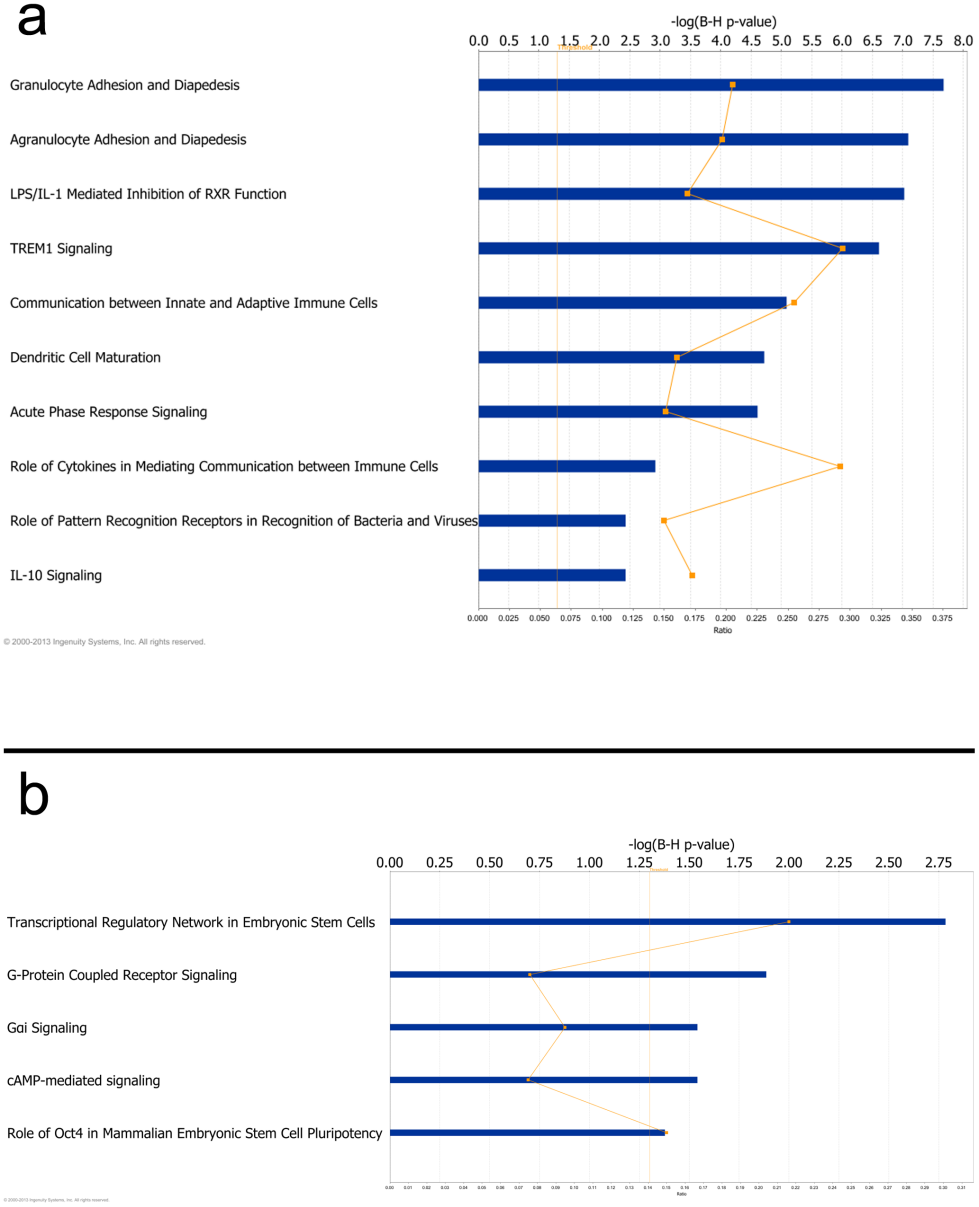
The most significant canonical pathways represented in our validated gene list. a. The horizontal blue bars showed the top10 significant canonical pathways that were altered epigenetically in the hypomethylated gene set, using Ingenuity Pathway Analysis (IPA). The orange square denotes the ratio of number of genes presented in our dataset over the total number of genes in that pathway. The top horizontal axis represents FDR (Benjamin-Hochberg) corrected P value, and the bottom one denotes ratio of number of genes presented in the dataset over the total number of genes. The vertical dotted line (in orange) represents the threshold of statistical significance. b. The 5 significant canonical pathways enriched in the hypermethylated gene set. Annotation is the same as the hypomethylated gene list.

IPA analyses were conducted separately for hypomethylated and hypermethylated genes. Our results showed that the top 10 significant canonical pathways involved in hypomethylation were all related to immune system functions (Figure 5a). In contrast, only 5 pathways were significant among hypermethylated genes (Figure 5b); the top one influenced embryonic stem-cell pluripotency, but other significant pathways were involved ubiquitously in cell signaling, such as cAMP and G-protein.

We then used the top two hypomethylated pathways, which are related to granulocyte and agranulocyte adhesion and diapedesis and created a pathway index as mentioned previously in the *Materials and Methods* section. This ***immune index*** included *CXCL10, C5AR1, CCL7, MYL4, ICAM2, IL1β, MMP14, SELE, IL18, IL1R1, MMP3, MMP19, CDH5, MYH4, ITGB2 and CCL11*. With the same method, we also created an index for stem cell pluripotency, which was the top hypermethylated pathway. The ***embryonic stem cell (EST) index*** included *GATA4, GATA6, NEUROG1, HOXB1, ISL1, FOXD3, GBX2 and MYF5*. These indices were later used in survival analyses (see below).

**Figure 6:**
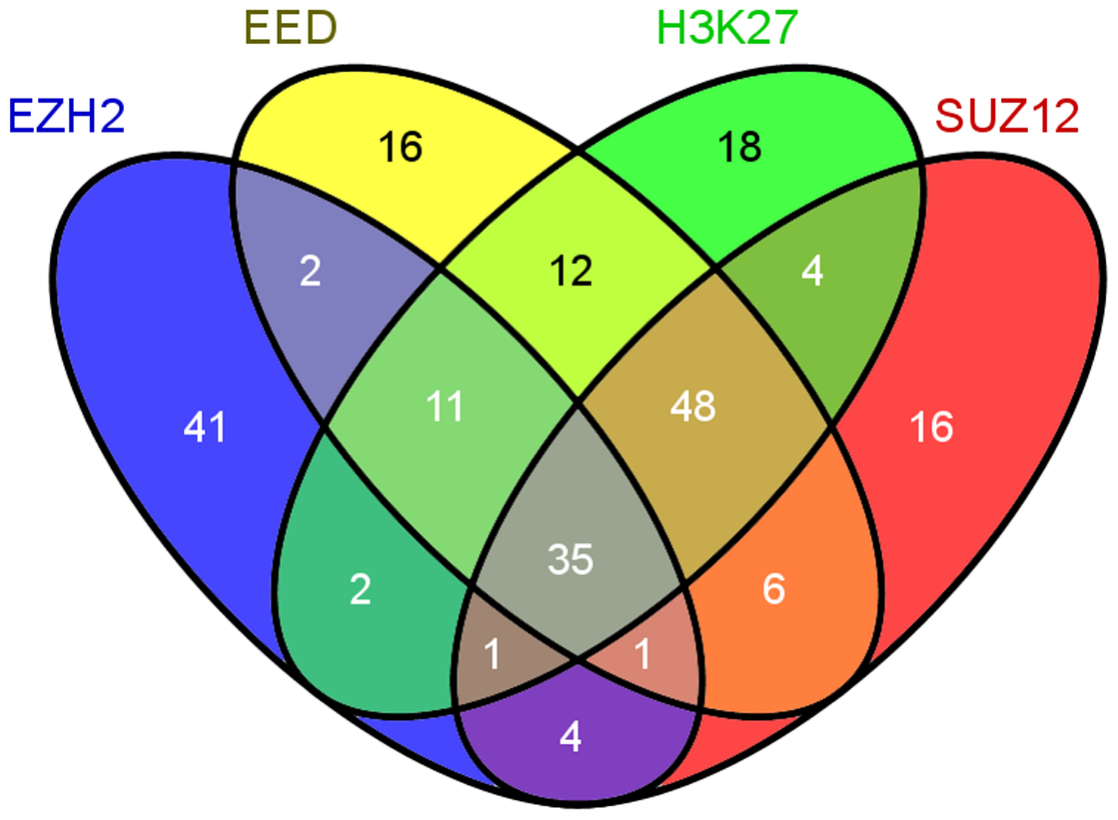
Enrichment of PRC2 and H3K27me^3^ in our validated gene list. Venn diagram showing the frequency of enrichment of PRC2 targets (*EZH2, SUZ12, EED*) and H3K27me^3^ in embryonic stem cells from our list of validated genes. The number of methylated genes for each enriched target and their overlaps were represented in corresponding areas inside the ellipses. Please note that overlapping areas are not drawn to scale.

We investigated targets of polycomb repressive complex 2 (PRC2) or histone 3 lysine 27 trimethylation (H3K27me^3^) in human embryonic stem cells (hESC). To quantify the degree of enrichment in our validated genes, we queried CHIP-seq datasets of H1-hESC (Tier 1) in ENCODE and from published papers [35], [36]. We downloaded lists of genes that are targets of H3K27me^3^ and PRC2, which included Suppressor of Zeste 12 Homolog (*SUZ12*), Embryonic Ectoderm Development (*EED*) and Enhancer of Zeste Homolog 2 (*EZH2*). The resulting lists of targets were matched to our validated gene list. Overall, 164 of 402 validated and hypermethylated genes (40.80%) were targets of at least one of PRC2 or H3K27me^3^, whereas 53 of 905 hypomethylated genes (5.86%) were their targets. Hypermethylated genes were enriched with PRC2 or H3K27me^3^ targets (χ^2^ = 245.42, df = 1, p = 0.0001). In total, 217 of 1307 genes (16.60%) were targets of PRC2 or H3K27me^3^. Figure 6 illustrates the frequency and overlap of enrichment of PRC2 and H3K27me^3^ in our validated, differentially methylated CpG sites using a Venn diagram.

### Correlation of Methylation Classes with Biomarkers of Global Methylation Levels

**Figure 7:**
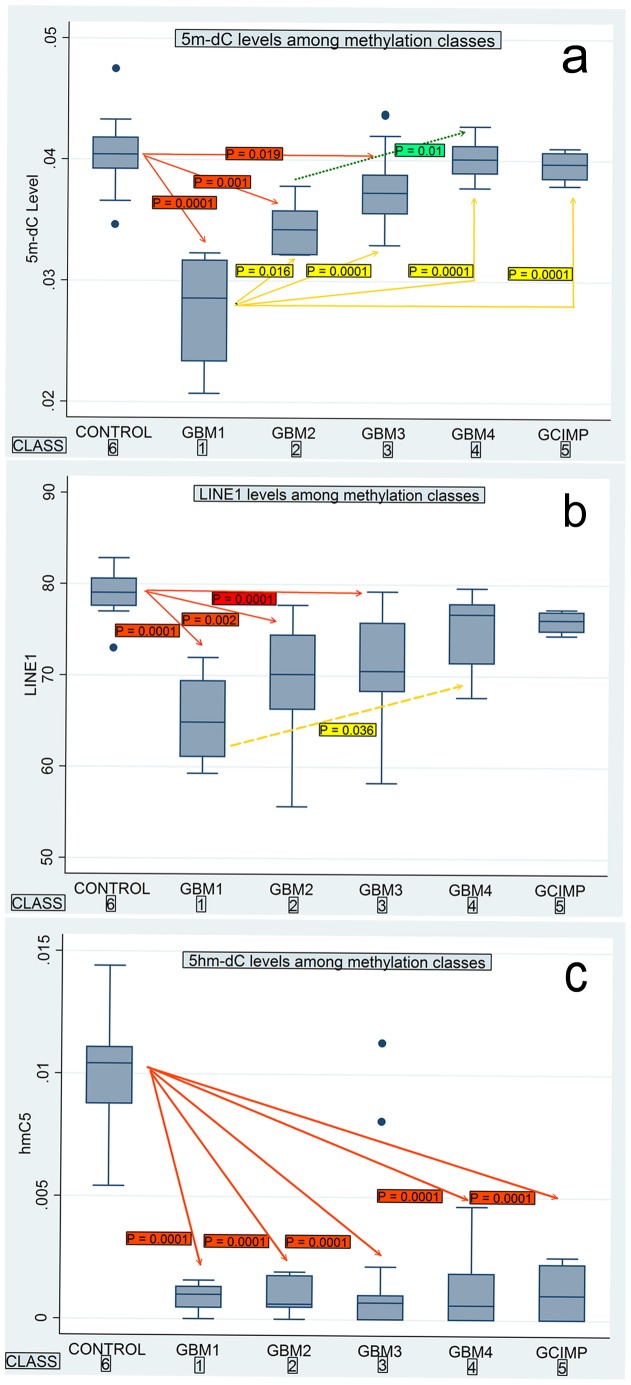
Levels of global methylation markers among methylation classes. a. Levels of 5m-dC between brain controls and tumors, and among methylation classes. Red, yellow and green lines (dotted) denoted pairwise comparison between two classes and the P values of their comparisons. b. Levels of LINE1 between brain controls and tumors, and among methylation classes. c. Levels of 5hm-dC between brain controls and tumors, and among methylation classes.

LINE1, 5m-dC and 5hm-dC levels were all significantly lower in GBMs compared to control brain tissues (p<0.0001 for all 3 markers). However, 5m-dC level was most capable in discriminating among various methylation classes (Figure 7a). In both 5m-dC and LINE1, global methylation levels were lowest in Class 1 tumor, and their levels successively rose from Class 1 to 5 (Figure 7a and 7b). Class 4 and 5 tumors had 5m-dC and LINE1 levels that were not statistically different from those of control brains. With respect to 5hm-dC levels, tumors were uniformly low compared to control tissues, but there were no differences in levels among tumor classes. (Figure 7c).

### Survival Analyses

Univariable and multivariable survival analyses results are shown in Table 5 and 6, respectively. Overall the median survival of the discovery population is 20.09 months (IQR: 9.11–34.39 months). In this dataset, age at diagnosis, KPS, study center (CWRU versus Columbia), LINE1 methylation level, *MGMT* methylation (50% methylated in control brains and 67% methylated in tumors), immune index and ESC index were all statistically significant prognostic factors in univariable analyses. Gross total resection and methylation Class 4 and 5 (*GCIMP*) showed trends towards favorable prognoses in the Univariable Cox models. When these variables were included in a multivariable Cox proportional hazards model, high level of LINE1 methylation (higher level of genomic stability), methylated *MGMT,* along with high KPS and gross total resection were all significant favorable prognostic factors. High levels of methylation in genes that affect stem cell pluripotency or promote differentiation, as indicated by higher score of the ESC index, remained a significant poor prognostic factor. However, age at diagnosis, *GCIMP*, study center and immune index were no longer significant in the presence of these other variables.

**Table 5:** Univariable Cox proportional hazard regression results of the discovery and validation datasets.

Discovery dataset			Validation dataset		
Factors	HR (95% CI)	P value	Factors	HR (95% CI)	P value
**Age at diagnosis**	1.03 (1.01–1.06)	0.006	**Age at diagnosis**	1.03 (1.02–1.05)	0.0001
**KPS**	0.94 (0.91–0.96)	0.0001	**KPS**	0.97 (0.95–0.98)	0.0001
**Surgery**			**Surgery**		
Biopsy	Reference		Biopsy	NA	
Subtotal resection	0.31 (0.068–1.40)	0.13	Subtotal resection	NA	
Gross total resection	0.28 (0.063–1.23)	0.093	Gross total resection	NA	
**Bevacizumab**			**Concomitant XRT/TMZ**		
None	Reference		No	Reference	
Received therapy	0.85 (0.46–1.55)	0.60	Yes	0.59 (0.40–0.86)	0.006
**LINE1 level**	0.93 (0.88–0.97)	0.003	**Bevacizumab**		
**5m-dC level**	0.75 (0.33–1.25)	0.34	No	Reference	
**5hm-dC level**	0.95 (0.58–4.40)	0.43	Yes	0.60 (0.40–0.91)	0.017
***MGMT*** ** methylation**			***MGMT*** ** methylation**		
Unmethylated	Reference		Cg12434587	0.43 (0.19–0.99)	0.048
Methylated	0.39 (0.21–0.75)	0.005	Cg12981137	0.46 (0.23–0.92)	0.028
**Methylation class**			**Methylation class**		
Class 3	Reference		Class 2	Reference	
Class 1	0.95 (0.33–2.73)	0.92	Class 1	0.59 (0.36–0.97)	0.039
Class 2	0.65 (0.23–1.86)	0.42	Class 3	0.59 (0.35–0.99)	0.048
Class 4	0.49 (0.21–1.14)	0.098	Class 4	0.81 (0.49–1.34)	0.41
Class 5 (*GCIMP*)	0.28 (0.066–1.19)	0.085	Class 5 (GCIMP)	0.21 (0.065–0.67)	0.009
**ESC index**	1.33 (1.04–1.69)	0.024	**ESC index**	3.91 (1.31–11.72)	0.015
**Immune index**	1.22 (1.07–1.38)	0.002	**Immune index**	2.04 (1.28–3.24)	0.003
**Participating Center**			**Participating Center**		
Columbia	Reference		Other 10 centers	Reference	
Case Western Reserve University	3.68 (1.89–7.18)	0.0001	Center 41	4.10 (1.86–9.04)	0.0001

**Table 6:** Multivariable Cox proportional hazard regression results of the discovery and validation datasets.

Discovery dataset		
Factors	HR (95% CI)	P value
**KPS**	0.94 (0.91–0.97)	0.0001
**Surgery**		
Biopsy	Reference	
Subtotal resection	0.20 (0.038–1.05)	0.058
Gross total resection	0.13 (0.025–0.73)	0.02
***MGMT*** ** methylation**		
Unmethylated	Reference	
Methylated	0.27 (0.13–0.60)	0.001
**LINE1 methylation**	0.95 (0.89–0.99)	0.048
**ESC index**	1.50 (1.17–1.91)	0.001
**Validation dataset**		
**Age at diagnosis**	1.03 (1.02–1.05)	0.0001
**KPS**	0.97 (0.96–0.99)	0.0001
**Concomitant XRT/TMZ**		
No	Reference	
Yes	0.53 (0.35–0.81)	0.003
**ESC index**	4.70 (1.45–15.16)	0.010
**Immune index**	2.23 (1.42–3.91)	0.005
**Participating Center**		
Other 10 centers	Reference	
Center 41	2.91 (1.24–6.84)	0.014
**Methylation class**		
Class 2	Reference	
Class 1	0.73 (0.40–1.33)	0.31
Class 3	0.46 (0.26–0.83)	0.010
Class 4	0.57 (0.32–1.01)	0.054
Class 5 (*GCIMP)*	2.22 (0.53–9.29)	0.28

We found 3 factors that were strongly associated with more advanced age at diagnoses and might have eliminated its effect in multivariable survival analyses: 1. A higher ESC index (p = 0.001); 2. A higher immune index, which denotes less hypomethylation in genes that control leukocyte trafficking (p = 0.032); 3. Low LINE1 methylation levels (p = 0.05). Due to our limited sample size and only 4. *GCIMP+* tumors, we were unable to confirm GCIMP’s prognostic ability. However, *GCIMP+* tumors showed strong correlation with MGMT methylation (p = 0.017).

The median survival of the TCGA validation dataset was 14.53 months (IQR: 7.63–21.30 months). Univariable Cox model showed that age at diagnosis, KPS, concomitant radiation with temozolomide (TMZ), treatment with Bevacizumab, study center 41, MGMT methylation, immune index and ESC index were all significant prognostic factors on their own. When the dominant Methylation Class 2 was served as a reference, Class 1, 3 and 5 (*GCIMP)* showed favorable prognoses, with *GCIMP+* group having the best prognosis. In the multivariable model, younger age at diagnosis, higher KPS, concomitant treatment with radiation and TMZ and methylation Class 3 were significant favorable prognostic factors. Higher scores of the immune index or less degree of hypomethylation in immunity related genes, high ESC index and study center 41 remained significant poor prognostic factors. Of note, methylation Class 5 (*GCIMP*) was no longer significant in the multivariable model. Instead, methylation Class 3 (with Class 2 as reference) showed a favorable survival after adjustment by other covariates.

When we evaluated factors that most influenced *GCIMP* status in logistic regression, younger age of onset and a lower score in the immune index (higher degree of hypomethylation) were the strongest factors associated with the *GCIMP+* group (p = 0.03 and p = 0.01, respectively). Thus, in multivariable survival analyses, these 2 factors had overshadowed *GCIMP* as more significant prognostic factors. Similarly, MGMT methylation level based on probes on the Illumina array was no longer a significant prognostic factor in the multivariable survival model. Again, high level of demethylation in immune related genes might have accounted for *MGMT’*s effect, as *MGMT* methylation was most strongly related to a lower score in the immune index (p = 0.007) and also *GCIMP+* status (0.01).

**Figure 8:**
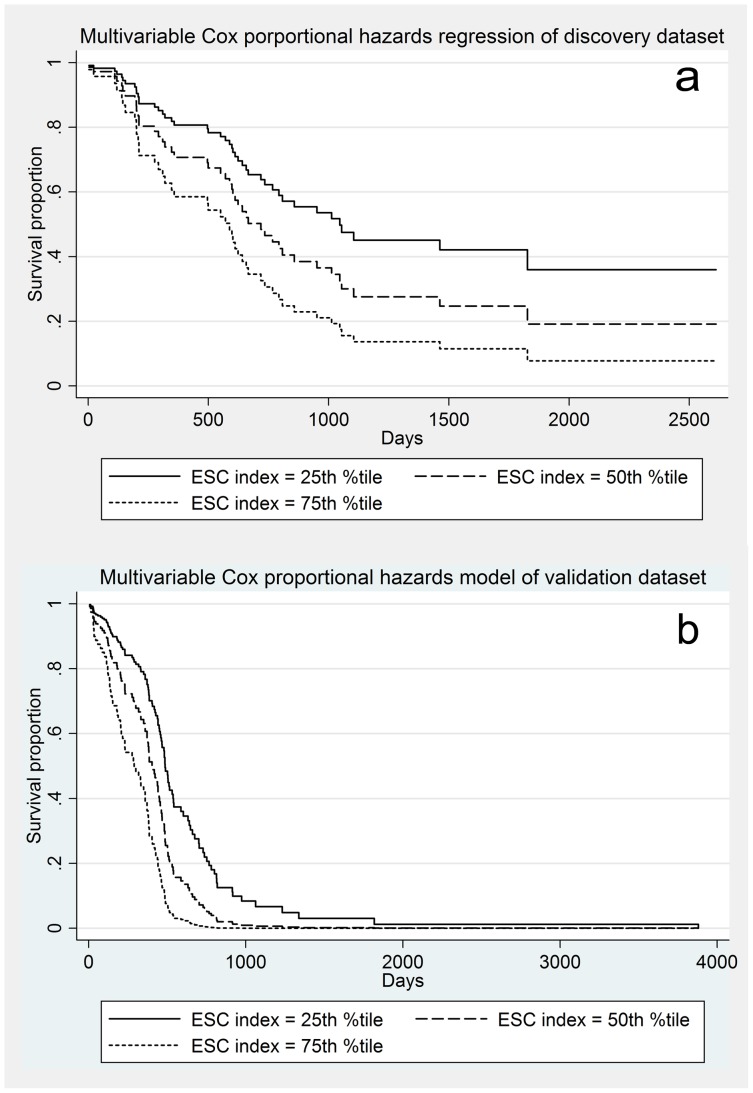
Adjusted Cox proportional hazard survival curves based on ESC index percentiles. a. Adjusted Cox survival curve of the discovery dataset, illustrating survival of subjects at the 25^th^, 50^th^ and 75^th^ percentiles of ESC index. b. Adjusted Cox survival curve of the validation dataset, illustrating survival of subjects at the 25^th^, 50^th^ and 75^th^ percentiles of ESC index.

Based on our analyses, the ESC index is a novel pathway-based biomarker for overall survival, and we were able to validate its prognostic significance in the TCGA dataset. Consistent between the two study populations, higher degree of methylation in genes that promote differentiation of stem cells is a poor prognostic factor. Figures 8a and 8b show the adjusted Cox survival curves based on EST index at 25^th^, 50^th^ and 75^th^ percentiles, for both the discovery (a) and validation cohorts (b). In both datasets, tests of proportional hazard assumptions on all covariates did not show any violation of this assumption.

## Discussion

This study validated more than 1500 differentially methylated sites and discovered 5 patterns of methylation changes across tumor samples in both the discovery and validation datasets. To our knowledge, it included the largest numbers of control brain tissues used for the investigation of differential methylation in glioma. The increase in brain control samples have helped us to better detect epigenetic alterations in de novo GBM. However, our PCA scatterplot illustrated that tumor methylation showed a wider amount of variability compared to the variability in controls. This finding may agree with that of another study that evaluated 139 cancer-specific differentially methylated regions (cDMRs) using a custom Illumina bead array. The investigator showed that differential methylation was characterized by increased stochastic variation in methylation level within each tumor type, suggesting a general disruption of the integrity of the cancer epigenome [33].

One feature of our differential methylation analyses is that there were more than twice as many hypomethylated CpGs as hypermethylated loci in the tumors. This finding is supported by differential methylation studies in other types of cancers, which suggested that hypomethylated loci are at least as numerous as, or often more abundant than hypermethylated CpGs [31]. However, hypomethylated loci, though more numerous, tended to show more moderate β changes compared to controls, whereas hypermethylated CpGs manifested larger changes even though they were fewer in numbers. A reason for this phenomenon may relate to differences in epigenetic remodeling, such as changes in chromatin marks, which lead to gene promoter hypomethylation versus those chromatin alterations that affect hypermethylation [37].

There were only 4 *GCIMP+*tumors and 5 *IDH1* mutant tumors in the discovery dataset. This supported the finding that *GCIMP* and *IDH1* mutations are uncommon findings in de novo GBM. In both datasets, strong correlation existed between methylated *MGMT,* or younger age of onset and *GCIMP+* status, which confirmed findings from previous investigations [2], [6]. However, hypermethylation relative to controls were found in discrete loci, or “blocks” and did not appear to be uniform across all CpGs. This may be due to the fact that brain controls were already hypermethylated in many CpG sites. Moreover, as one recent systematic review pointed out, there has been a lack of consensus on the precise definition of *CIMP* in cancers [38].

For genes that were differentially methylated in our dataset, we were able to demonstrate that different pathways are involved in hypo- and hypermethylated genes. We found genes that regulate the immune system, affecting both innate and cellular immunities, were aberrantly hypomethylated in the tumors. Previous publications had focused primarily on gene hypermethylation; thus this finding will hopefully prompt future studies on how immune pathways, through epigenetic alterations, will relate to the generation of immune-suppressive tumor environment, or the host’s ability to detect and eliminate GBM.

Our IPA canonical pathway results showed different pathways were affected by hypermethylation. The top pathway confirmed that epigenetic regulation was crucial to maintenance of stem cell pluoripotency in GBM. Related to this finding is that almost 40% of hypermethylated CpG sites were targets of PRC2 or H3K27me^3^. PRC2 was up-regulated in glioblastoma stem-like cells [39]. PRC2 targeted developmentally important genes, induced compact chromatin, repressed expression of target genes and maintained “stemness” in embryonic stem cells [40]. These same genes were targets of hypermethylation in cancer, via transformation from a polycomb-dependent silencing to methylation-dependent silencing during cancer development. Moreover, it appeared that the DNA demethylating agent, 5-deoxy-aza-cytidine (DAC) was able to reverse methylation and induce gene expression in cancer cells that were marked by both repressive chromatin marks (positive for H3K27me^3^) and methylation, but histone deacetylase inhibitor (HDACi) was not able to re-activate these genes [1], [36]. These results indicated that hypermethylated genes in cancer, even if they were maintained in a suppressed state by polycomb marking, are competent to reactivate upon removal of the methylation mark. Nevertheless, in GBM, demethylating agents have not been considered in clinical trials because promoter *MGMT* methylation is related to temozolomide (TMZ) response [41]. Moreover, in view of the fact the genes involved in immune system functions were hypomethylated in their promoters, and repetitive elements of de-methylation may trigger further genomic instability, broad-spectrum, demethylating drugs may potentially bring on undesirable consequences and genomic instability.

Consistent with findings from other cancers, GBM also showed global hypomethylation when compared to control tissues. Our three biomarkers consistently demonstrated that tumors were hypomethylated compared to control brains. Since these markers measured cytosine methylation across compartments in the human genome, and repetitive elements consisted of >65% of genomic CpG sites versus 5.5% of them in promoters of protein coding genes, demethylation in the repetitive elements may contribute to a significant degree of global hypomethylation in GBM compared to control tissues [42]. Nevertheless, since LINE1 and 5m-dC levels in methylation Class 4 and Class 5 (*GCIMP*) were similar to controls, the epigenetic alterations of these tumors might involve a lesser degree of wide-spread, concomitant de-methylation of the repetitive elements. Future studies using bisulfite next gen sequencing will help to further define boundaries between hypo-and hypermethylated domains in the GBM epigenome, as one study had recently done in other cancer types [33].

The reasons for the strong and uniform depletion of 5hm-dC in GBM were not clear. *TET1*, which converts 5m-dC to 5hm-dC, has not been shown to be mutated or over-expressed in GBM. Although it is also a global methylation marker and is closely related to 5m-dC, 5hm-dC is enriched within the gene body, promoters and transcription start site, whereas 5m-dC and LINE1 were primarily located in heterochromatin and repetitive elements [43]. In the evaluation of prognostic marker, LINE1, was a significant prognostic factor for overall survival in the discovery dataset, even after it was adjusted for the effect of other known prognostic factors such as MGMT methylation, extent of surgical resection and KPS. This marker is worthy of further validation in larger study or clinical trial, as the TCGA does not have information on this biomarker.

We were able to show that higher ESC index score was a poor survival factor in the discovery dataset, and the TCGA dataset validated its prognostic significance. Thus, high levels of methylation of genes involved in this pathway may maintain these cells in the pluripotent state and promote treatment resistance. However, therapeutic design to re-differentiate this gene set cannot be “off-the shelf” demethylating agents. The approach will have to target other epigenetic components that promote these methylation markers, or specific enhancers/suppressors of these genes.

Future studies may also want to elucidate factors that lead to methylation changes in GBM, such as alteration in chromatin states, and local DNA features. Some studies have suggested that the presence of SINE and LINE may predispose methylation spread into nearby gene promoters, unless certain insular DNA elements, such as the presence of *CTCF*, *SP1* or *USF1*, protect against such spreading into downstream promoters [44]–[46]. This study illustrated that repetitive element demethylation and epigenetic alteration in gene promoters occur hand-in-hand, but whether destabilization of repetitive elements may enhance methylation spread into adjacent genes in GBM will need further laboratory evaluation.

## Supporting Information

Figure S1 **Heat map showing unsupervised hierarchical clustering using** ***GCIMP*** **markers.** a. Using GCIMP markers, four GBM subjects were found to be *GCIMP+* in the discovery dataset (red lines on the row dendrogram). They corresponded to 4 of the 5 IDH1 mutated subjects. b. Using *GCIMP* markers, 13 GBM subjects were found to be *GCIMP+* in the validation dataset (red lines on the row dendrogram). Six of them had IDH1 mutations.
(TIF)

Figure S2 **Top down views of PCA analyses.** a. In the discovery dataset, the separate, posterior location of the GCIMP+ group was best appreciated from this view; b. In the validation dataset, GCIMP+ group occupied a similar location posteriorly. Also, segregation of the 2 control groups was better visualized in this view.
(TIF)

Table S1 **List of pyrosequencing primers used in pyrosequencing validation studies.**
(PDF)

Table S2 **The complete list of 1864 differentially methylated CpG sites, their median β difference and statistical significance levels from our discovery dataset.**
(XLSX)

Table S3 **The complete list of 2452 differentially methylated CpG sites, their median β difference and statistical significance levels from our TCGA validation dataset.**
(XLSX)

Table S4 **A list of 1548 CpG probes and associated gene names that were differentially methylated in both the discovery and validation datasets.**
(PDF)
