# Supplementary figures and images for: Genome-Wide Methylation Analyses in Glioblastoma Multiforme
Source: PLoS One. 2014 Feb 21;9(2):e89376. doi: 10.1371/journal.pone.0089376 (PMC3931727; doi:10.1371/journal.pone.0089376)

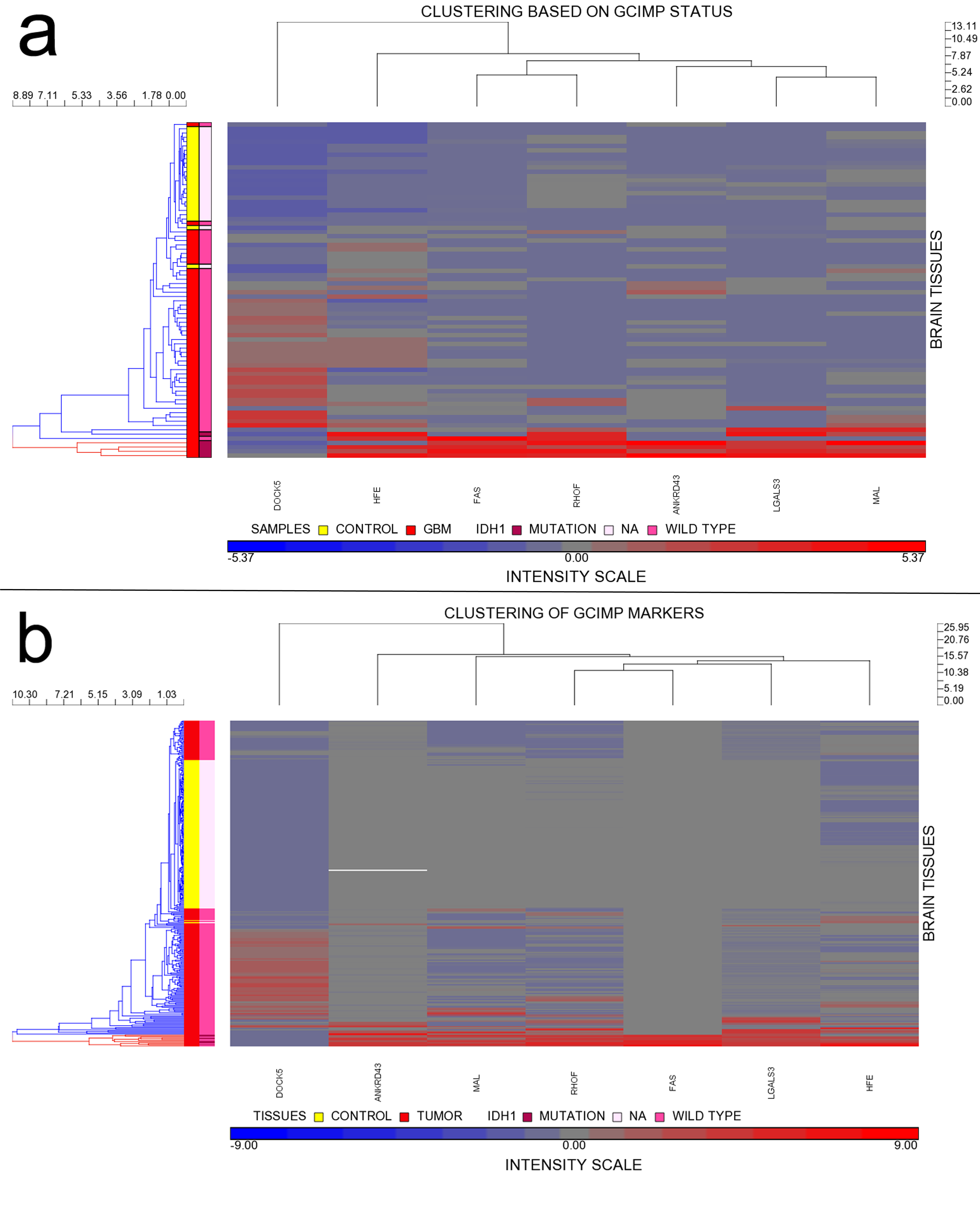

Supplement: Figure S1 — Heat map showing unsupervised hierarchical clustering using GCIMP markers. a. Using GCIMP markers, four GBM subjects were found to be GCIMP+ in the discovery dataset (red lines on the row dendrogram). They corresponded to 4 of the 5 IDH1 mutated subjects. b. Using GCIMP markers, 13 GBM subjects were found to be GCIMP+ in the validation dataset (red lines on the row dendrogram). Six of them had IDH1 mutations. (TIF) [file pone.0089376.s001.tif]

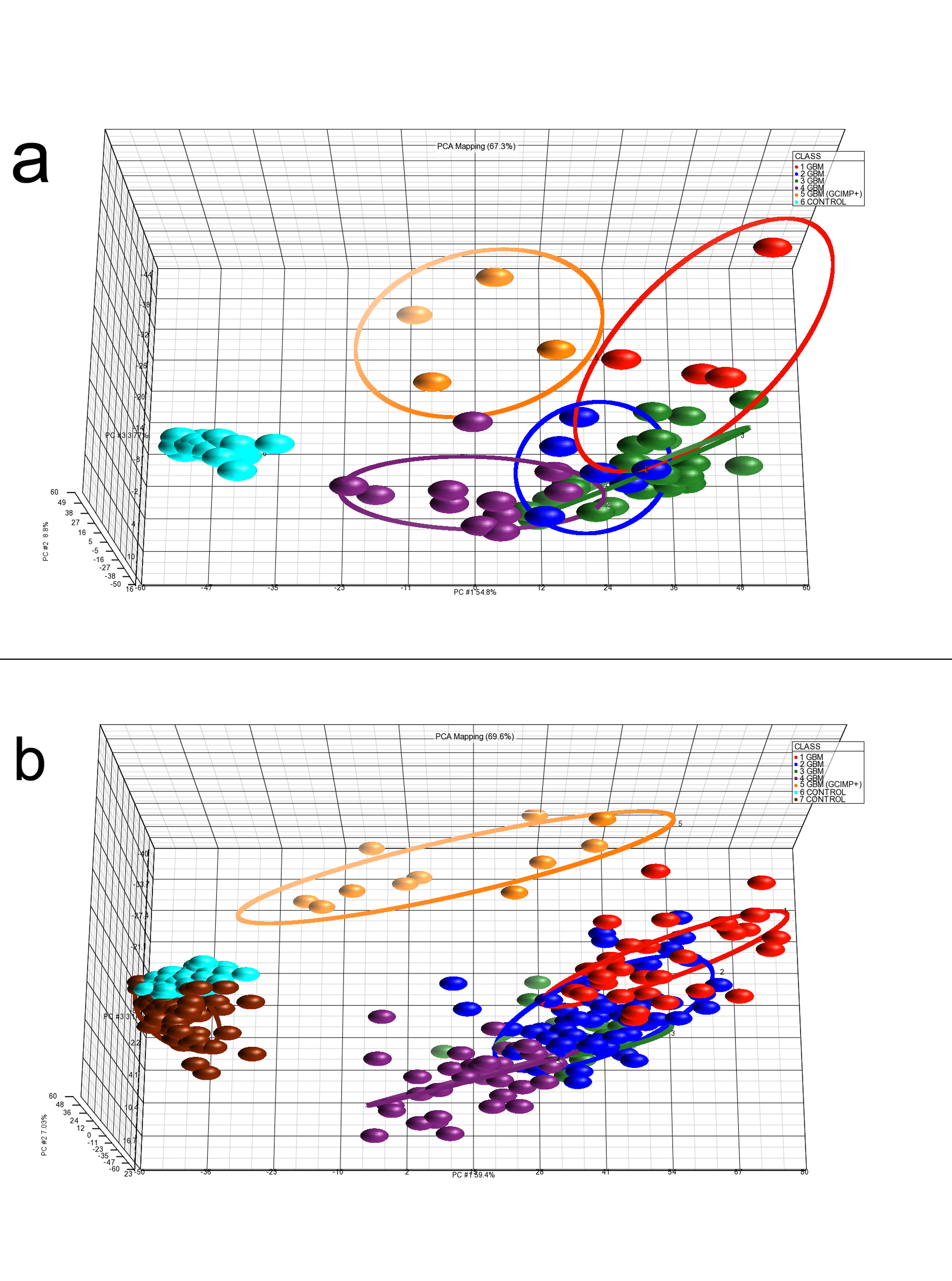

Supplement: Figure S2 — Top down views of PCA analyses. a. In the discovery dataset, the separate, posterior location of the GCIMP+ group was best appreciated from this view; b. In the validation dataset, GCIMP+ group occupied a similar location posteriorly. Also, segregation of the 2 control groups was better visualized in this view. (TIF) [file pone.0089376.s002.tif]
