# Supplementary material for: Genome-Wide Methylation Analyses in Glioblastoma Multiforme
Source: PLoS One. 2014 Feb 21;9(2):e89376. doi: 10.1371/journal.pone.0089376 (PMC3931727; doi:10.1371/journal.pone.0089376)
Supplement: Table S1 — List of pyrosequencing primers used in pyrosequencing validation studies. (PDF) [file pone.0089376.s003.pdf]

CpG site of genes	Technical conditions and primers used
BST2_cg01254505	95°C_15min, 45 x [94°C_30s, 56°C_30sec, 72°C_30sec], 72°C_10min. Amplicon -130 bp. Final primer conc. 0.2uM. Bisulfite treated DNA template - 1ul. Primers: forward: TAGGGGAGGGTGTTGGAAT ; reverse: TAATCCTCTACCTATAAATACCTCATACA (5'biotin) ; sequencing: GGGTTATTTTTTTATTAGTATAG. Sequence to analyze: GAAAYGTTTTGATTAATAGTAAGTTTTAGGA .
DAB2IP_cg13060154	95°C_15min, 45 x [94°C_30s, 56°C_30sec, 72°C_30sec], 72°C_10min. Amplicon - 175bp. Final primer conc. 0.2uM. Bisulfite treated DNA template - 1ul. Primers: forward: GGGGTTAGGTGAGTAGAAGAGA ; reverse: ATCCTAACCACAAAAACCCCACTT (5' biotin) ; sequencing: GAGGAGAGTAGAGGG. Sequence to analyze: TGTTYGYGGYGGTTYGGGYGAGGTYGGGYGYGAAGTTTTTYGTAGGGAAGTTTTGT.
PCDHGB4_cg26282384	95°C_15min, 45 x [94°C_30s, 56°C_30sec, 72°C_30sec], 72°C_10min. Amplicon -99bp. Final primer conc. 0.2uM. Bisulfite treated DNA template - 1ul. Primers: forward: ATTGTATTGGTTAATTTGAAGTAGTAGAG; reverse: AAATCTAAAACCCCAAACTCTCCACTT (5'biotin) ; sequencing: GTTGTAGTTTTTTAGTGTTGA. Sequence to analyze: TTTTGGGYGTYGTTGTTGGTTAAAGTGGAGAGTTTG.
DGKE_ cg1344452	95°C_15min, 45 x [94°C_30s, 56°C_30sec, 72°C_30sec], 72°C_10min. Amplicon -92bp. Final primer conc. 0.2uM. Bisulfite treated DNA template - 1ul. Primers: forward: AAGGGGGAGGGGAAGGGA ; reverse: CCTCTCCCCTTCCATCTTCTCC (5'biotin) ; sequencing: TGTTTTTTTTTTGGTTAGGTA . Sequence to analyze: TYGTTTTTGGAGAAGATGGAAG.
BHMT_cg10660256	95°C_15min, 45 x [94°C_30s, 56°C_30sec, 72°C_30sec], 72°C_10min. Amplicon -258 bp. Final primer conc. 0.2uM. Bisulfite treated DNA template - 1ul. Primers: forward: ATAGGAGGAGGGTTTGATGAAGG (5'biotin) ; reverse: ATCCCCTAAAAACTCACCTTCTTAAC ; sequencing: CTTCCTAATATCCAAACAAATAATC. Sequence to analyze: RACACRAATACRAACCRACTAAAC.
