# Supplementary material for: Genome-Wide Methylation Analyses in Glioblastoma Multiforme
Source: PLoS One. 2014 Feb 21;9(2):e89376. doi: 10.1371/journal.pone.0089376 (PMC3931727; doi:10.1371/journal.pone.0089376)
Supplement: Table S4 — A list of 1548 CpG probes and associated gene names that were differentially methylated in both the discovery and validation datasets. (PDF) [file pone.0089376.s006.pdf]

id	genes
cg00718513	
cg13279585	
cg23984130	
cg25159668	AADACL2
cg00295325	ABCB11
cg20025970	ABCC9
cg14982472	ABCG1
cg18328933	ABHD14A
cg05488632	ABHD9
cg25839227	ABI3
cg05064181	ABLIM1
cg14401592	ACCN5
cg02812142	ACMSD
cg18766847	ACMSD
cg26780333	ACOT4
cg07153965	ACRV1
cg27087809	ACSBG1
cg27257408	ACSBG1
cg13619990	ACSM1
cg10078415	ACSM3
cg02998425	ADAM21
cg05997860	ADAM21
cg05847038	ADAM7
cg15610233	ADAM7
cg14143055	ADAMDEC1
cg13059335	ADAMDEC1
cg04601137	ADAMTSL5
cg13878010	ADCY5
cg22461835	ADRA1A
cg17963840	ADRA1A
cg21542793	ADRA2B
cg10235817	ADRA2C
cg24426405	AGR2
cg19125606	AGT
cg16967583	AGXT
cg11166252	AHR
cg11003133	AIM2
cg10636246	AIM2
cg20959866	AJAP1
cg07639198	AKR1C1
cg22074467	AKR1C2
cg09277575	AKR1D1
cg03365437	ALDH1A2
cg19177941	ALDH1A3
cg07730301	ALDH3B1
cg09533063	ALDH8A1

cg00577167	ALDOB
cg18809289	ALOX5
cg11052143	ALS2CR11
cg07376232	AMICA1
cg02100629	AMID
cg26540515	ANGPT4
cg22150335	ANK3
cg12354377	ANK3
cg09533293	ANXA2
cg15831515	ANXA3
cg22792910	ANXA4
cg14451276	AOAH
cg21602160	AOC3
cg19264571	APCDD1
cg26353877	APCS
cg04570669	APIN
cg15727249	APOA4
cg26692016	APOBEC1
cg22375610	APOBEC2
cg22954818	APOBEC3A
cg07186138	APOBEC3C
cg04286933	APOBEC3G
cg13119609	APOC2
cg07713361	APOL1
cg03128832	APOL5
cg20713492	AQP10
cg01359534	AQP10
cg19220825	AQP5
cg07327347	AQP8
cg01418124	ARHGAP24
cg09954385	ARHGAP8
cg14265075	ARHGEF11
cg16792160	ASAH2
cg21038703	ASB16
cg17041296	ASB17
cg06263495	ASCL2
cg09245073	ASGR1
cg12951282	ASGR2
cg05820087	ATP13A4
cg06123346	ATP4A
cg06811800	ATP4B
cg01248426	ATP6V0D2
cg12958813	ATP6V1G3
cg12111714	ATP8A2
cg18236477	ATP8A2
cg18085435	ATP8B1
cg15001381	AXIN1

cg16176379	AYTL1
cg12019109	AZGP1
cg00332153	BANK1
cg17241310	BARHL2
cg20910746	BBOX1
cg02609880	BCAN
cg08927738	BCAS1
cg05386606	BCL7B
cg10528989	BDKRB1
cg10238171	BDKRB1
cg05890484	BHMT
cg03400060	BHMT2
cg23571857	BIRC4BP
cg11308639	BMP10
cg18952647	BNC1
cg19988449	BNC1
cg17560332	BOLL
cg12879425	BRDG1
cg16313343	BRF1
cg25391023	BTNL2
cg24024214	BTNL8
cg05886367	BTNL9
cg25414165	C10orf11
cg11204562	C10orf81
cg07621046	C10orf82
cg17349199	C10orf82
cg22045288	C10orf91
cg13853761	C11orf2
cg07747336	C11orf38
cg02477931	C11orf39
cg14738823	C12orf46
cg13682722	C14orf102
cg20103550	C14orf155
cg27398547	C14orf39
cg08278554	C15orf48
cg18490846	C17orf73
cg18017908	C17orf76
cg11393848	C1QC
cg20483374	C1QTNF5
cg15776355	C1R
cg01471713	C1orf115
cg05767404	C1orf150
cg18839416	C1orf158
cg06007645	C1orf175
cg00030047	C1orf188
cg08623383	C1orf38
cg11750883	C1orf42

cg08460435	C1orf59
cg08253405	C1orf62
cg22726338	C1orf62
cg09851465	C1orf87
cg12278770	C1orf90
cg08527127	C1orf94
cg11319389	C20orf100
cg04369341	C20orf100
cg13236107	C20orf152
cg24691461	C20orf160
cg21519900	C20orf186
cg06661994	C20orf195
cg01671881	C20orf71
cg01869233	C20orf75
cg27132814	C20orf79
cg21663722	C20orf91
cg06940107	C21orf100
cg10198932	C21orf129
cg25864727	C21orf42
cg23732182	C21orf84
cg09238677	C3AR1
cg24389347	C3orf22
cg22959932	C3orf32
cg17803430	C4BPA
cg11070419	C4BPA
cg05659947	C4BPB
cg10585462	C4orf7
cg25600236	C4orf7
cg02149446	C5AR1
cg14722162	C5orf20
cg11976616	C6
cg13281868	C6orf142
cg13258700	C7orf13
cg07671603	C7orf13
cg22199118	C8orf34
cg04721098	CACNG3
cg06226384	CACNG5
cg18705776	CALML3
cg17792192	CAMSAP1
cg26937500	CARD11
cg02516189	CARD9
cg20798152	CART
cg02409351	CART1
cg01999333	CASP14
cg09243021	CASP2
cg17453778	CASR
cg14894216	CATSPER1

cg13745346	CBFA2T3
cg21902544	CBLN2
cg23124451	CBX7
cg27585441	CCDC54
cg07177852	CCDC68
cg12071073	CCDC70
cg20556988	CCL1
cg17118262	CCL1
cg24870391	CCL11
cg09256683	CCL14
cg26548883	CCL15
cg10190509	CCL16
cg08214029	CCL18
cg27443224	CCL21
cg11207564	CCL3L3
cg25659818	CCL4
cg12455187	CCL5
cg02936263	CCL7
cg01636591	CCL8
cg27000831	CCL8
cg16794682	CCND1
cg04111761	CCR3
cg13615963	CCR6
cg13504059	CCR7
cg09033997	CCR9
cg25358289	CD14
cg07264679	CD163
cg07950803	CD1A
cg04574507	CD1B
cg13765621	CD1D
cg09478478	CD1E
cg12200412	CD1E
cg16719404	CD2
cg17638468	CD200R1
cg07608333	CD209
cg01618851	CD209
cg11939496	CD244
cg04995095	CD300E
cg10523494	CD300LB
cg15374234	CD300LF
cg11122968	CD33
cg18508525	CD36
cg09554443	CD3Z
cg05200628	CD48
cg13311440	CD48
cg10516886	CD53
cg12971694	CD72

cg04790874	CD79A
cg05921699	CD79A
cg16776350	CD84
cg02945019	CD84
cg13164309	CDA
cg00750606	CDA
cg26185508	CDCP2
cg17655614	CDH1
cg24765079	CDH1
cg15175266	CDH12
cg20987610	CDH17
cg27043873	CDH18
cg22319147	CDH5
cg23181133	CEACAM3
cg21529807	CEACAM4
cg21126943	CEACAM6
cg19623751	CEACAM7
cg08551633	CEACAM8
cg22815534	CECR5
cg06268694	CELSR1
cg07926025	CER1
cg01474260	CESK1
cg07443748	CESK1
cg23557926	CFH
cg12687463	CFHR1
cg09551916	CFHR2
cg03192551	CGI-69
cg07423149	CHI3L1
cg26366091	CHI3L2
cg07484827	CHRNA10
cg04953015	CHRNA2
cg22563815	CHRNA5
cg07906724	CHRNA6
cg12827555	CHX10
cg09424896	CIB3
cg15364618	CIDEB
cg01351032	CIITA
cg10313673	CILP2
cg19154438	CKM
cg07173760	CLC
cg22181664	CLCA1
cg17306637	CLDN20
cg15544036	CLDN4
cg27190239	CLEC2A
cg01120761	CLEC4C
cg14162076	CLEC4D
cg21372914	CLEC4M

cg01532771	CLEC4M
cg26066361	CLEC7A
cg26823505	CLPS
cg06933965	CMKLR1
cg19000186	CNGA1
cg26151675	CNR2
cg10503138	CNTN4
cg16254309	CNTNAP2
cg16907566	COL14A1
cg13553455	COL17A1
cg01796223	CPA4
cg07156669	CPD
cg03258472	CRB3
cg06336792	CREM
cg22152192	CRYM
cg07260017	CSF1R
cg09585781	CSH2
cg19216731	CSRP3
cg06489008	CST11
cg10423860	CST5
cg02882813	CST5
cg15210427	CST9L
cg26952662	CTHRC1
cg15043057	CTPS
cg24355048	CTSG
cg03887787	CTSW
cg27504991	CUL7
cg04569233	CX3CR1
cg25806808	CXCL1
cg01288089	CXCL10
cg17001652	CXCL13
cg25432696	CXCL6
cg22670329	CXCL6
cg03826976	CYB5R2
cg07136161	CYLC2
cg04968473	CYP1A2
cg07905963	CYP2A13
cg25427638	CYP2A7
cg26822241	CYP2C9
cg11267879	CYP2F1
cg23326197	CYP3A4
cg23414387	CYP4B1
cg03190825	CYP4F11
cg05358291	CYP4F2
cg27067618	CYP4F3
cg10751811	CYP4Z1
cg10238818	CYYR1

cg14837165	D4ST1
cg13060154	DAB2IP
cg25406518	DAK
cg18552413	DARC
cg17470143	DCAMKL1
cg13396068	DCD
cg25372195	DCD
cg04515001	DCDC2
cg16306115	DCDC2
cg00077877	DDEF1
cg15875314	DDX4
cg10758292	DEFA1
cg17267907	DEFA1
cg04747619	DEFA5
cg19033555	DEFB1
cg25214366	DEFB103A
cg17288121	DEFB103A
cg05810550	DEFB106A
cg20312687	DEFB118
cg18462653	DEFB119
cg20305726	DEFB126
cg02046532	DEFB129
cg22478614	DEFB4
cg01344452	DGKE
cg04623955	DIO3
cg00933411	DLC1
cg09873258	DLK1
cg27016494	DLX5
cg20080624	DLX5
cg13462129	DLX5
cg20120491	DMC1
cg13191049	DMN
cg00250430	DMRT2
cg11530960	DMRT2
cg02067021	DNAJC5B
cg21233722	DOCK2
cg25192419	DOCK5
cg04836038	DOCK9
cg03732056	DOK2
cg14386061	DOK2
cg10303487	DPYS
cg09936561	DRD5
cg15439862	DSC3
cg11832722	DSC3
cg17233935	DSCR10
cg17142149	DSCR1L1
cg01337047	DSG1

cg22386311	DSG1
cg24711914	DSG4
cg13445249	DSG4
cg14563260	EDG2
cg06501070	EDG7
cg16264526	EDNRA
cg27513764	EFCAB3
cg05037688	EGFL7
cg13481359	EGR4
cg13084525	EHF
cg21685266	EHHADH
cg01963696	ELA2
cg27069753	ELA3B
cg05822532	ELN
cg02727423	EML1
cg25277950	EML2
cg08831348	EML2
cg03096975	EML2
cg01795122	EMP3
cg22889448	EMR1
cg07072643	EMR3
cg15746620	EMR3
cg15552238	EMR3
cg21051046	EN2
cg24910675	ENG
cg00027083	EPB41L3
cg18431127	EPB42
cg18997129	EPHA1
cg27105123	EPS8L1
cg07115820	EPX
cg03127334	ERG
cg08972170	Ells1
cg24529858	F11
cg06570843	FABP2
cg19904463	FABP5
cg06640279	FAIM3
cg12285118	FAM107B
cg01623187	FAM112A
cg11398517	FAM112A
cg12376406	FAM12B
cg13921352	FAM19A4
cg16352283	FAM46B
cg10106284	FAM49A
cg04282622	FAM71C
cg05761971	FAM82A
cg03954858	FAM83F
cg00221494	FARP1

cg00201234	FBLN2
cg25084878	FBN2
cg01420388	FBXO2
cg14696870	FCER1A
cg03221619	FCER2
cg19103704	FCGBP
cg27470554	FCGR2A
cg22436411	FCGR2B
cg04384208	FCGR3A
cg15761405	FCRL2
cg25259754	FCRL3
cg08786003	FCRL3
cg01612158	FCRL4
cg11241627	FERD3L
cg25691167	FERD3L
cg03782727	FFAR1
cg02017155	FFAR3
cg21624359	FFAR3
cg12827188	FGA
cg13726191	FGFBP1
cg01593385	FGG
cg19145398	FKHL18
cg13447818	FLG
cg03602500	FLJ00060
cg09001953	FLJ11200
cg18006568	FLJ12056
cg00745735	FLJ14437
cg25484904	FLJ21511
cg01432087	FLJ21749
cg18216249	FLJ21908
cg07425555	FLJ23447
cg22960952	FLJ23657
cg05215575	FLJ25410
cg00044729	FLJ25421
cg24169915	FLJ25773
cg25569462	FLJ25801
cg24743310	FLJ31196
cg02245378	FLJ32447
cg25596297	FLJ32447
cg14159672	FLJ32569
cg11521325	FLJ32926
cg12491659	FLJ33641
cg26331247	FLJ33706
cg17774418	FLJ33708
cg20678353	FLJ35695
cg27449489	FLJ35767
cg10412312	FLJ36004

cg22262140	FLJ36046
cg04086012	FLJ36180
cg03819692	FLJ36701
cg08626653	FLJ37538
cg00970385	FLJ38725
cg10525488	FLJ39370
cg21930712	FLJ40235
cg07409200	FLJ40919
cg01720228	FLJ42461
cg08489623	FLJ44060
cg25032865	FLJ44186
cg09848074	FLJ44186
cg13897627	FLJ44674
cg18354594	FLJ45717
cg02255732	FLJ46154
cg12682367	FLJ46358
cg03167883	FLJ46365
cg25093045	FLJ46481
cg03782453	FLJ90575
cg25044651	FLJ90650
cg15489294	FLJ90650
cg07017374	FLT3
cg03764585	FMOD
cg26987645	FMOD
cg00325491	FN5
cg04848046	FNDC3B
cg17574251	FNDC8
cg03699566	FOLR1
cg11295113	FOLR2
cg07676849	FOLR3
cg11260422	FOXA1
cg22815110	FOXD3
cg18815943	FOXE3
cg15811427	FPR1
cg05376954	FPR1
cg24964368	FPRL1
cg06784466	FPRL2
cg16176600	FRK
cg25902889	FSD1
cg27420123	FSHB
cg13881341	FUT1
cg14719352	FUT1
cg14173523	FUT5
cg20692569	FZD9
cg26673195	G6PC
cg08099701	GABRA5
cg24387380	GABRA5

cg22672790	GABRA6
cg15739581	GALNT3
cg25310567	GALNTL5
cg21402035	GALR3
cg22975712	GALR3
cg24922045	GAS2L2
cg10016608	GAS2L2
cg26420196	GAS6
cg26020513	GATA4
cg01546563	GATA4
cg09626984	GATA4
cg20279283	GATA4
cg11981599	GATA4
cg25216696	GATA4
cg13434842	GATA4
cg19172575	GATA4
cg24646414	GATA4
cg19496782	GATA6
cg22074858	GBP3
cg06214007	GBP6
cg23095584	GBX2
cg07376029	GC
cg25462303	GCET2
cg20022122	GCKR
cg02844545	GCM2
cg22289360	GCNT1
cg02255004	GDEP
cg02774160	GGT1
cg18538812	GIF
cg17738194	GK2
cg17099569	GLI2
cg14679587	GLS2
cg00057593	GML
cg01565918	GNAS
cg09649610	GNG4
cg19238840	GP2
cg18691434	GPC2
cg09069593	GPR114
cg23855093	GPR128
cg26514492	GPR132
cg08375941	GPR15
cg18741908	GPR160
cg21870884	GPR25
cg22631938	GPR27
cg23661676	GPR42
cg04754011	GPR44
cg02965078	GPR44

cg26252167	GPR6
cg08072716	GPR62
cg10189695	GPR78
cg06776256	GPRC5A
cg04033774	GPSM2
cg13283751	GPX5
cg26170660	GPX5
cg23276115	GRAP
cg16806597	GRAP
cg03840259	GRAP2
cg21296230	GREM1
cg14859460	GRM6
cg15674997	GRM6
cg09868882	GRM8
cg04340502	GSTA3
cg25567232	GSTA5
cg02091100	GUCA1A
cg19561186	GUCA1C
cg13131015	GUCY2C
cg14425294	GUCY2D
cg00407150	GUP1
cg13143729	GYPE
cg03875678	GZMB
cg02150910	GZMH
cg22228134	GZMH
cg01550148	H2AFY
cg18833140	HABP2
cg01580681	HAND2
cg19110684	HAVCR2
cg14544583	HBB
cg10920765	HBG2
cg18499731	HBII-438B
cg21960110	HBZ
cg24468890	HDAC1
cg05446471	HDAC11
cg15820955	HDAC7A
cg24127874	HES6
cg10608341	HESX1
cg25462291	HEYL
cg07136254	HFE2
cg06589885	HFE2
cg11378840	HHEX
cg07022477	HIF3A
cg25274750	HIPK2
cg14652095	HIST1H1A
cg05554718	HIST1H1D
cg10046620	HIST1H2AI

cg03221914	HIST1H2AJ
cg07922606	HIST1H3E
cg02599464	HIST1H4I
cg11037148	HIST1H4J
cg00634577	HIST1H4K
cg10608333	HIST1H4K
cg13906813	HLA-DPA1
cg22282941	HLA-DQA2
cg16899306	HLA-DQB2
cg19717150	HNF4A
cg15760840	HOXA11
cg17950095	HOXA11
cg09871315	HOXA2
cg26069745	HOXA2
cg26511321	HOXA7
cg25047280	HOXA9
cg27009703	HOXA9
cg01381846	HOXA9
cg07778029	HOXA9
cg07823492	HOXB1
cg25882366	HOXB2
cg08089301	HOXB4
cg15539420	HOXB8
cg22709192	HOXC11
cg03918304	HOXD10
cg21591742	HOXD10
cg16632715	HOXD11
cg03874199	HOXD12
cg23130254	HOXD12
cg00005847	HOXD3
cg18702197	HOXD3
cg01152019	HOXD4
cg12127282	HOXD4
cg00767581	HOXD4
cg18750960	HOXD4
cg15520279	HOXD8
cg21815667	HOXD8
cg14991487	HOXD9
cg10957151	HOXD9
cg02506908	HPD
cg09584711	HPR
cg11547724	HPX
cg08815403	HSD17B13
cg16175792	HSD3B1
cg09458237	HSPA12B
cg16319578	HSPA2
cg27120999	HSPA2

cg18740800	HSPCAL3
cg25040282	HTN3
cg11990309	HTR1E
cg06096336	HTR2B
cg24134767	HTR3A
cg06531741	HTR3B
cg19815720	HTR3C
cg15835825	HTR5A
cg05663262	HYI
cg25041439	HYLS1
cg15583072	IAPP
cg20034100	IBRDC1
cg07918509	ICAM2
cg08090640	IFI35
cg16823701	IFNA1
cg01713938	IFNA13
cg01906717	IFNA13
cg06479216	IFNA16
cg13235447	IFNA2
cg07236769	IFNW1
cg06638433	IGF2BP1
cg03876618	IGFBP7
cg25623640	IGJ
cg11304234	IL18
cg18156583	IL18RAP
cg12380764	IL19
cg07935264	IL1B
cg05949173	IL1F10
cg10269439	IL1F7
cg02229946	IL1F7
cg10479672	IL1F8
cg26759925	IL1F9
cg15233681	IL1R1
cg16386158	IL1RL1
cg11916609	IL1RL1
cg00136405	IL21
cg02656594	IL21R
cg25946374	IL22RA1
cg06796611	IL24
cg11270633	IL28B
cg27412902	IL29
cg11733245	IL2RA
cg23813257	IL32
cg04312209	IL7R
cg13519373	IL8RA
cg09617773	IMPG1
cg25764191	INA

cg20664201	INHBE
cg13993218	INS
cg25336198	INS
cg10691387	IQCF2
cg14940420	IQCF2
cg17534899	IQSEC1
cg24826867	IRF8
cg15433631	IRX2
cg03963198	IRX4
cg02833725	ISG20L2
cg21410991	ISL1
cg26896762	ISL1
cg26233914	ITGAX
cg24167037	ITGB2
cg08374799	ITGB7
cg22909609	ITGBL1
cg09453312	ITK
cg08356693	ITLN1
cg05440289	IVL
cg10234985	IZUMO1
cg07265310	K5B
cg17255302	K6HF
cg08126211	KAAG1
cg08832227	KCNA1
cg27409364	KCNC1
cg06572160	KCNC3
cg05675373	KCNC4
cg27553955	KCNG3
cg27320127	KCNK12
cg02611419	KCNK17
cg25229305	KCNK18
cg01495509	KCNMB1
cg12949760	KCNQ1
cg01530101	KCNQ1DN
cg05373457	KCNS2
cg18239753	KHDRBS2
cg13105904	KIAA0323
cg00292971	KIAA0773
cg09088834	KIAA0980
cg17729667	KIAA0980
cg05477024	KIAA1804
cg13234863	KIAA1944
cg24654350	KIR3DL1
cg20176989	KIR3DL2
cg11237817	KIR3DL3
cg23282559	KL
cg03667047	KL

cg11220060	KLF1
cg02983451	KLF11
cg04528819	KLF14
cg20523861	KLHL1
cg03907363	KLK1
cg26415633	KLK1
cg09226684	KLK10
cg06130787	KLK10
cg09254939	KLK10
cg09702010	KLK11
cg07947016	KLK2
cg16742703	KLK3
cg04349727	KLK5
cg17031773	KLK9
cg13995453	KLRB1
cg00443307	KLRG1
cg10742225	KRT13
cg13350783	KRT13
cg01602596	KRT14
cg23128056	KRT15
cg27478659	KRT16
cg24457403	KRT16
cg27236973	KRT17
cg06378617	KRT23
cg22392708	KRT23
cg02784874	KRT9
cg02579133	KRTAP10-8
cg07014174	KRTAP11-1
cg22643217	KRTAP11-1
cg02764897	KRTAP13-1
cg16431978	KRTAP13-3
cg14062083	KRTAP13-4
cg16812893	KRTAP15-1
cg15606663	KRTAP15-1
cg00948500	KRTAP20-2
cg22373097	KRTAP21-1
cg06222800	KRTAP21-1
cg23581186	KRTAP21-2
cg18822544	KRTAP26-1
cg23205633	KRTAP4-2
cg11618577	KRTCAP3
cg08611714	KRTHA2
cg15983005	KRTHA3A
cg14533138	KRTHA3B
cg25545210	KRTHA4
cg02780988	KRTHA6
cg18848394	KRTHA8

cg07643942	LACRT
cg10125195	LACRT
cg04000821	LAIR2
cg01726767	LALBA
cg14894144	LAMA3
cg01318557	LAT2
cg03375002	LAT2
cg12022621	LAX1
cg14696820	LCE1A
cg08878744	LCE1B
cg24304714	LCE1C
cg15531099	LCE1D
cg21065959	LCE1E
cg23413307	LCE1F
cg21754343	LCE2B
cg25098401	LCE2B
cg00406188	LCE2C
cg21312148	LCE2D
cg03741352	LCE3C
cg20676475	LCE3D
cg23110514	LCE3E
cg21846488	LCE4A
cg17542385	LCE4A
cg01868128	LCE5A
cg23815000	LCN1
cg11873854	LCN6
cg14611112	LCN6
cg08300860	LDB3
cg21783004	LECT2
cg22462235	LEFTY2
cg20661303	LEFTY2
cg19594666	LEP
cg11081833	LGALS2
cg03082060	LGALS8
cg26545162	LGICZ1
cg08468689	LGP1
cg12351433	LHCGR
cg19486673	LILRA2
cg00705255	LILRA3
cg13733733	LILRA3
cg01204985	LILRA4
cg20542190	LILRA4
cg05248470	LILRB2
cg24447890	LMAN1L
cg09358725	LMO2
cg22227965	LMO7
cg05461841	LOC124220

cg26687173	LOC126248
cg02046017	LOC220070
cg11608424	LOC253012
cg22131172	LOC283487
cg00546897	LOC284837
cg01605783	LOC284837
cg09039163	LOC339768
cg21745164	LOC63928
cg15309006	LOC63928
cg17761453	LOR
cg07260592	LPA
cg12032049	LPO
cg24489015	LPO
cg26632776	LRRC3
cg07753583	LRRC61
cg06641366	LRRC8C
cg04151683	LRRN6C
cg10995925	LTA
cg25775449	LTB4R
cg20542800	LTB4R
cg11394785	LTC4S
cg16361890	LTC4S
cg08569678	LY6K
cg01367992	LY9
cg23732024	LY96
cg12768605	LYPD5
cg16097772	LYZ
cg14912034	LYZL2
cg21044104	LYZL4
cg13986618	M160
cg01615704	MALL
cg11009736	MARCO
cg01078434	MAS1L
cg20725021	MASP1
cg13882988	MBL2
cg27418851	MBL2
cg20801056	MBP
cg06836736	ME1
cg24881834	ME1
cg04291079	MEG3
cg16019620	MEP1A
cg01888566	MEST
cg09059945	MEST
cg09872616	MEST
cg08077673	MEST
cg17277939	MGC13168
cg14117297	MGC23244

cg18750756	MGC29671
cg24642468	MGC33367
cg17699374	MGC35206
cg00892393	MGC35212
cg17199658	MGC39715
cg06627364	MGC4677
cg00411097	MGC9712
cg19680672	MGMT
cg14129786	MGMT
cg13302154	MGP
cg02620013	MLNR
cg07935568	MLNR
cg20795913	MLX
cg05316065	MLZE
cg09208010	MMP14
cg16725130	MMP19
cg12493906	MMP26
cg16466334	MMP3
cg25119415	MNDA
cg05598246	MOG
cg10585962	MOGAT2
cg04988978	MPO
cg09421562	MPO
cg13181019	MPP7
cg10431340	MPZ
cg23001457	MRGPRX1
cg24252809	MRGPRX1
cg13062935	MRGPRX2
cg16446783	MRGPRX4
cg17959722	MRPL41
cg13765961	MS4A1
cg06806711	MS4A1
cg23307338	MS4A12
cg22197708	MS4A2
cg17173423	MS4A3
cg25944100	MS4A3
cg04353769	MS4A6A
cg05040360	MS4A6E
cg06269753	MSC
cg23710218	MSC
cg08244028	MSH3
cg01668126	MSR1
cg16303562	MSR1
cg20588069	MSX1
cg27096144	MSX2
cg26796283	MSX2
cg15842276	MTNR1B

cg18939260	MTSS1
cg21130374	MX2
cg26207503	MYF5
cg00134787	MYH1
cg23400451	MYH4
cg21301148	MYH6
cg10591174	MYL2
cg05294455	MYL4
cg18176712	MYL5
cg01514487	MYO1A
cg09666654	MYO1B
cg08441170	MYO3A
cg23771603	MYO3A
cg22077553	MYOC
cg14600885	MYOZ3
cg18484189	NALP10
cg03789934	NALP11
cg02347487	NALP14
cg26264314	NALP5
cg22190114	NALP8
cg12639234	NAT2
cg08116137	NAT8
cg09172980	NAT8
cg18267374	NEF3
cg02994956	NEFH
cg26186727	NETO1
cg04330449	NEUROG1
cg14958635	NEUROG1
cg17568996	NFAM1
cg07986525	NFE2L3
cg08247612	NHLH1
cg00772000	NHLRC1
cg22736354	NHLRC1
cg21428681	NKX3-1
cg08109815	NMBR
cg09632136	NNMT
cg14209518	NNMT
cg04799664	NOD27
cg23896545	NOMO1
cg21006686	NOS1
cg14700707	NOTCH4
cg17754680	NPC1L1
cg20202438	NPPA
cg00548268	NPTX2
cg12799895	NPTX2
cg05158615	NPY
cg12614105	NPY

cg16762386	NR0B2
cg02554564	NTF3
cg04740359	NTF3
cg22721827	NTN2L
cg23555120	NUAK1
cg16140179	NURIT
cg23101680	NURIT
cg19789466	OAS1
cg20870559	OAS2
cg15538820	OBP2B
cg20789824	OLFML2A
cg07829804	OLR1
cg02250594	ONECUT2
cg20906802	OPN5
cg03898365	OR10A4
cg22951794	OR10A5
cg25076881	OR10J1
cg21414251	OR12D2
cg20856834	OR12D3
cg16678925	OR1A2
cg27429194	OR1A2
cg02721374	OR1D2
cg27622610	OR1G1
cg06882926	OR1G1
cg11884699	OR2A4
cg12351042	OR2B2
cg08634024	OR2F1
cg20507276	OR2L13
cg05779068	OR2W1
cg06353345	OR51B4
cg25890048	OR5I1
cg03780486	OR6A2
cg06639544	OR7A5
cg02124291	OR7A5
cg06131936	OSBPL5
cg14511156	OSCAR
cg06637774	P2RY6
cg05671350	P4HA3
cg05590982	P8
cg15149645	P8
cg23228178	PADI4
cg03885639	PANX3
cg10994126	PAPPA2
cg07937272	PARP12
cg19345602	PATE
cg08886154	PAX4
cg00509670	PAX9

cg26620157	PAX9
cg26717133	PCDH21
cg07899016	PCDHB12
cg11368643	PCDHB15
cg10757144	PCDHB15
cg11864993	PCDHB16
cg02260587	PCDHB2
cg14011639	PCDHGB7
cg23563234	PCDHGB7
cg02797569	PCOLCE
cg26777475	PCOLCE
cg20249919	PCSK6
cg07713493	PCTK3
cg07211259	PDCD1LG2
cg13899108	PDE4C
cg17861230	PDE4C
cg12435792	PDE6B
cg19635695	PDE6C
cg11297236	PDILT
cg23696886	PDLIM2
cg11245384	PDYN
cg04598121	PENK
cg06403553	PGK2
cg06275635	PGLYRP3
cg09448880	PGLYRP3
cg01987509	PGR
cg08013810	PHF20
cg17783509	PHOX2B
cg24719601	PHOX2B
cg04893119	PI15
cg09462575	PI3
cg02105856	PIGR
cg26628847	PIP
cg26368842	PIP5K2A
cg06144905	PIPOX
cg24495017	PITX1
cg14056644	PITX2
cg26215428	PKD2
cg11377136	PKDREJ
cg18885346	PKHD1
cg09871043	PKHD1
cg21985470	PKLR
cg02510853	PKMYT1
cg19103609	PKN1
cg09009380	PKP1
cg01851399	PKP2
cg18133966	PLA2G1B

cg15228639	PLA2G4E
cg16543027	PLCB2
cg27609819	PLCL1
cg02833180	PLCL1
cg25573386	PLCZ1
cg06339706	PLEKHA4
cg21832150	PLEKHN1
cg14003512	PLGLB2
cg03075662	PLSCR2
cg22549408	PMAIP1
cg12530080	PMCHL1
cg16028753	PODN
cg16739580	POP2
cg19868730	POPDC2
cg10874403	POU1F1
cg04632671	PPARG
cg26866325	PPBPL2
cg04375036	PPP1CC
cg00967316	PPP1R3A
cg24727203	PPP1R3B
cg15765694	PPP3R2
cg12374721	PRAC
cg09196942	PRAMEF1
cg03891191	PRAMEF2
cg27345534	PRB2
cg14076161	PRB4
cg16799087	PRDM7
cg17342283	PRG1
cg15357945	PRG2
cg24459209	PRG3
cg12626411	PRG4
cg05436658	PRKCB1
cg18959478	PRKCDBP
cg18392783	PRKCDBP
cg18828334	PRKD2
cg14149007	PRKD2
cg15748507	PRLHR
cg17397493	PRLR
cg27404050	PRODH2
cg02741177	PROL1
cg06585690	PROL1
cg09595479	PRPH
cg12878228	PRSS1
cg26504906	PRSS16
cg20839025	PRSS7
cg07073964	PRSSL1
cg21450627	PSD4

cg27257987	PSG4
cg19873701	PSG9
cg24989962	PTGDR
cg11546621	PTGDS
cg06738602	PTGER2
cg15201877	PTGER3
cg17683775	PTGES
cg24816298	PTH
cg03391568	PTHR1
cg25057743	PTHR2
cg09863066	PVALB
cg23412777	PYGO1
cg19884600	PYHIN1
cg01714932	PZP
cg12448933	RAB37
cg10399228	RAG1
cg15982419	RALBP1
cg16872071	RALGDS
cg03085312	RARA
cg09952204	RASGRF2
cg24310246	RAXL1
cg13099330	RBP1
cg17964955	RBP3
cg02192520	RDH5
cg17243643	RDH5
cg13573276	RDHE2
cg23111544	REG1A
cg24240626	REG3A
cg17923358	RELN
cg05163071	RETNLB
cg15303841	RFPL1
cg00406844	RFPL3
cg20979799	RFXDC1
cg13614181	RGC32
cg02148642	RGPD5
cg04041960	RGS10
cg06025017	RGSL2
cg19862344	RHAG
cg10523019	RHBDD1
cg18180155	RIBC2
cg00594952	RIMS3
cg17016000	RIN2
cg10318258	RIPK3
cg00722300	RLN3
cg13718960	RNASE1
cg26191951	RNASE2
cg24851490	RNASE2

cg07525077	RNASE3
cg15910079	RNASE3
cg22828602	RNF133
cg12293634	RNF149
cg04994456	RNF186
cg16008138	RNF190
cg25766046	ROR2
cg11854007	RP11-49G10.8
cg12936220	RPL26L1
cg07693270	RPL39L
cg01797043	RPL3L
cg16612562	RRP22
cg00689340	RTKN
cg00117172	RUNX3
cg06377278	RUNX3
cg19764418	RYR2
cg10795646	S100A10
cg02813121	S100A12
cg23499956	S100A16
cg13997435	S100A2
cg24898863	S100A8
cg15484375	SAA1
cg22587758	SAA4
cg06303238	SALL4
cg27160701	SBEM
cg17981339	SBEM
cg05106502	SCAP1
cg01772980	SCGB1D1
cg10848367	SCGB1D2
cg14472601	SCGB3A1
cg22421766	SCNN1D
cg01857260	SCRL
cg18294257	SEC14L3
cg09426307	SEC14L3
cg20436912	SEC14L4
cg11119884	SEC63D1
cg09784259	SELE
cg07640473	SEMA3F
cg04545516	SEMG1
cg02311163	SEMG2
cg03552103	SEPT10
cg24621042	SERPINA1
cg19937039	SERPINA10
cg19042947	SERPINA4
cg09147827	SERPINA6
cg27056119	SERPINB1
cg11435943	SERPINB12

cg03468463	SERPINB12
cg13943564	SERPINB2
cg10533434	SERPINB3
cg15422147	SERPINB5
cg00226904	SERPINB5
cg08411049	SERPINB5
cg01568736	SERPINB7
cg03380645	SFTPB
cg03600318	SFTPD
cg26104469	SGCZ
cg26920757	SH2D3C
cg21283680	SH3BP5
cg14898892	SHRM
cg03283694	SIGLEC11
cg12530021	SIGLEC12
cg01193293	SIGLEC7
cg23458892	SIGLEC7
cg04164824	SIGLEC9
cg13407883	SIGLEC9
cg02672220	SIM2
cg11061975	SIRPB2
cg15722679	SIRPB2
cg17423978	SIRPD
cg06092815	SKIP
cg00714377	SLA2
cg18881723	SLAMF1
cg07837085	SLAMF7
cg11721194	SLAMF7
cg18312429	SLC10A2
cg22971191	SLC10A2
cg18854666	SLC11A1
cg07719512	SLC11A1
cg22752533	SLC12A5
cg22040627	SLC13A5
cg16652063	SLC13A5
cg26980692	SLC15A3
cg21992250	SLC15A3
cg19777783	SLC15A4
cg03835296	SLC17A1
cg15916061	SLC17A4
cg06836849	SLC17A8
cg22628926	SLC25A11
cg04996020	SLC26A3
cg07103493	SLC27A6
cg05521696	SLC2A14
cg23338195	SLC30A8
cg06501790	SLC34A1

cg21200703	SLC34A2
cg19616230	SLC34A2
cg25437385	SLC35F3
cg10345936	SLC36A2
cg01678321	SLC36A3
cg02192965	SLC3A1
cg21495715	SLC5A10
cg13473336	SLC5A2
cg16232126	SLC5A7
cg10141715	SLC5A8
cg03064067	SLC6A15
cg02064402	SLC6A18
cg19421752	SLC6A18
cg10084993	SLC9A3R2
cg00995065	SLCO1B1
cg27104271	SLCO2B1
cg18972811	SLIT2
cg12966875	SLPI
cg07441143	SLURP1
cg21948655	SMCP
cg15239123	SMOC1
cg10979891	SMOC1
cg19297232	SMPD3
cg10556064	SMPD3
cg00891541	SMPD3
cg17217677	SMPD3
cg08113203	SMR3B
cg02288165	SN
cg00893242	SND1
cg19803984	SNRPN
cg20179697	SNX9
cg26845300	SNX9
cg10784813	SOCS1
cg06630241	SOCS2
cg06614002	SOX10
cg02919422	SOX17
cg26829529	SPACA3
cg11161417	SPACA3
cg19787037	SPAG11
cg25107903	SPAG11
cg25802093	SPAG6
cg22724153	SPAM1
cg09022993	SPATA18
cg02423618	SPATA8
cg04786857	SPDY1
cg06147863	SPI1
cg01917648	SPIC

cg17788013	SPINK5
cg26851800	SPINK5L2
cg23765993	SPINLW1
cg10379687	SPINLW1
cg15375239	SPINT2
cg04505023	SPRR1A
cg06101324	SPRR1A
cg18780284	SPRR1B
cg24884084	SPRR1B
cg18766755	SPRR2A
cg26059632	SPRR2A
cg12891678	SPRR2D
cg14826683	SPRR2D
cg00152644	SPRR2E
cg04138756	SPRR3
cg08763351	SPRR4
cg02202484	SPRR4
cg22730004	SPTA1
cg19502744	SRD5A2
cg15403517	SRD5A2
cg02164046	SST
cg27590397	SSTR1
cg10019507	SSTR4
cg15503752	ST6GALNAC1
cg03001305	STAT5A
cg00436282	STATH
cg18320336	STEAP1
cg00564163	STEAP4
cg00616129	STYK1
cg13968390	SULT1C1
cg00698688	SULT2B1
cg21053323	SUMO3
cg18087477	SYCP1
cg02608019	SYK
cg10025443	SYK
cg05801648	SYK
cg15873301	SYN2
cg05342835	SYNC1
cg17339202	SYNC1
cg15824056	SYNJ2
cg00902195	SYT10
cg23950724	SYT10
cg21604615	SYTL1
cg14221171	TAC1
cg14924278	TACC2
cg04263186	TACR3
cg16076328	TACSTD1

cg16080552	TACSTD2
cg05262335	TAGAP
cg19797376	TAL1
cg03465320	TAP1
cg16853860	TAP1
cg23248452	TAS2R1
cg04901273	TBC1D3
cg23350580	TBC1D3C
cg02601403	TBC1D3C
cg06747888	TBR1
cg13870866	TBX20
cg02008154	TBX20
cg18536148	TBX4
cg10281002	TBX5
cg21907579	TBX5
cg23131007	TCF12
cg24215443	TCF21
cg00187686	TCN1
cg20018806	TCN1
cg09656934	TDRD5
cg12277666	TDRD5
cg07613153	TDRD9
cg14252059	TECTA
cg00453193	TES
cg24621354	TES
cg21491308	TEX101
cg19418958	TEX15
cg20939319	TEX15
cg25593948	TFAP2B
cg11158374	TFF2
cg04806409	TFF3
cg18081104	THEM5
cg21303011	THRB
cg18994063	TIMD4
cg12397274	TINAG
cg27090087	TINAG
cg27022827	TJP3
cg07195577	TLCD1
cg15915418	TLE1
cg05429895	TLR4
cg22484793	TLR9
cg25720804	TLX3
cg25942450	TLX3
cg06800962	TM4SF1
cg04121771	TM4SF4
cg20277416	TM7SF2
cg00447208	TMC8

cg03998348	TMCO5
cg23254045	TMCO5
cg04482110	TMEM106A
cg02089348	TMEM129
cg20955688	TMEM71
cg27159719	TMEM71
cg19686152	TMOD3
cg19510180	TMPRSS11B
cg06399881	TMPRSS11B
cg03536003	TMPRSS11D
cg20695587	TMPRSS11F
cg11826486	TMSL3
cg11947493	TNFRSF10D
cg22783363	TNFRSF10D
cg10964421	TNFRSF10D
cg01031400	TNFRSF10D
cg06495803	TNFRSF7
cg08840010	TNFRSF9
cg21094154	TNFSF11
cg10791260	TNFSF15
cg01186777	TNFSF9
cg21295911	TNFSF9
cg07189381	TNNT1
cg13980719	TNP1
cg13823701	TNXB
cg14494812	TOB1
cg08965143	TP53I3
cg00565688	TP73
cg16607065	TP73
cg06196379	TREM1
cg26928682	TREML2
cg13663218	TRHDE
cg22268164	TRHR
cg06051311	TRIM15
cg27091343	TRIM15
cg15238224	TRIM31
cg22502502	TRIM38
cg07405796	TRIM40
cg09196959	TRIM40
cg02284188	TRIM49
cg03882305	TRIM50C
cg20855565	TRIM58
cg13998293	TRIM63
cg06493386	TRPA1
cg01610488	TRPA1
cg08191854	TRPM2
cg02786019	TRPV6

cg16752583	TRPV6
cg10466917	TRY1
cg14153740	TRY1
cg13975369	TSGA14
cg09058542	TSHB
cg15747595	TSPYL5
cg08901867	TTLL6
cg04008843	TTR
cg06437862	TUBA2
cg12073537	TUBA6
cg07307078	TUBB6
cg23435746	TWSG1
cg02600394	TXK
cg20981615	TXK
cg03417466	TYR
cg07326586	UBD
cg03167763	UBXD3
cg06911113	UBXD3
cg10135708	UCN2
cg23338993	UGT1A6
cg10432859	UGT1A7
cg19399100	UGT2A1
cg12374577	UGT2A3
cg23183296	UGT2B28
cg03498559	UGT2B4
cg04558553	UGT2B7
cg19147390	UHRF1
cg12324629	ULK2
cg07456201	UMOD
cg25013053	UNC45B
cg09682183	UNC93A
cg10725344	UNQ1940
cg12315311	UNQ3033
cg05241571	UNQ467
cg10305797	UNQ467
cg17536071	UNQ689
cg17162024	UNQ9433
cg21289015	USH1C
cg12954718	USP6
cg12374431	VAX2
cg04609640	VMD2L2
cg02184413	VNN1
cg18441959	VPREB1
cg26093711	VRK2
cg15309264	VRK2
cg07054641	WDR52
cg15044041	WDR52

cg00690280	WFDC10B
cg25133753	WFDC11
cg20485165	WFDC12
cg08108953	WFDC13
cg09874776	WFDC13
cg00980978	WFDC2
cg24765446	WFDC6
cg22021786	WFDC8
cg12061127	WFDC9
cg00240880	WISP2
cg19718882	WIT-1
cg20616414	WNK2
cg01830294	WNT2
cg04096767	WT1
cg04456238	WT1
cg15446391	WT1
cg16463460	WT1
cg01693350	WT1
cg12006284	WT1
cg06516124	WT1
cg13301003	WT1
cg13641903	WT1
cg22533573	WT1
cg16092786	WT1
cg16501028	WT1
cg05222924	WT1
cg03584220	XCL2
cg12286890	XCL2
cg25298754	ZBED2
cg10133777	ZD52F10
cg24641352	ZFP41
cg12680609	ZFP41
cg06274159	ZFP42
cg06566994	ZNF167
cg05250458	ZNF177
cg09643544	ZNF177
cg20880234	ZNF198
cg00899659	ZNF22
cg18680834	ZNF536
cg03975694	ZNF540
cg27389185	ZNF540
cg05221167	ZNF560
cg08109646	ZNF683
cg02440177	ZNF702
cg03872376	ZP4
